# Modeling and Experimental Study of Cross-Electromechanical Admittance for Crack Localization in Beams with Distributed Piezoelectric Wafers

**DOI:** 10.3390/s26196216

**Published:** 2026-09-30

**Authors:** Lijun Yang, Wei Yan

**Affiliations:** School of Civil Engineering and Future Cities, Ningbo University, Ningbo 315211, China; ylj9909@126.com

**Keywords:** crack localization, cross-electromechanical admittance (EMA), transfer matrix method (TMM), steel specimens, correlation coefficient (CC)

## Abstract

This research developed a novel crack localization technique that employed cross-electromechanical admittance (EMA), and its validity was verified through both analytical simulations and experimental measurements. Initially, an analytical model of the cross-EMA was established based on the transfer matrix method (TMM), and subsequently verified by experimental data and finite element analysis. Using the established model, the cross-EMA signatures were then utilized for crack localization in conjunction with a damage index defined as the correlation coefficient (CC). Furthermore, experimental investigations were carried out on five steel specimens, each of which was instrumented with multiple surface-bonded Lead Zirconate Titanate (PZT) wafers in a distributed manner. A crack with a width of 1.5 mm and a depth of 8 mm was introduced into each specimen to simulate structural damage. Both analytical investigations and experimental research have demonstrated that the cross-EMA signatures exhibit crack localization capabilities and can precisely reflect the local mechanical characteristics of the monitored structure.

## 1. Introduction

As the primary load-bearing components in engineering structures, beams play critical roles in determining the structural integrity, safety, and service life of entire structural systems. However, during long-term service, beam members will inevitably be affected by loads, environmental corrosion, and other factors, resulting in cracks, corrosion, and other damages. If these damages are not detected and repaired in time, they may lead to structural failure and even serious safety accidents. Therefore, the development of efficient and accurate damage identification methods has important theoretical significance and engineering application value. With increasingly complex and large-scale engineering structures, some traditional detection approaches—such as laser scanning [1], optical fiber sensing [2], acoustic emission monitoring [3], and radar detection [4]—have difficulty meeting the needs of early identification and location of minor damages, especially when applied to environmentally challenging conditions and localized damage quantification.

In the mid-1990s, a novel electromechanical admittance (EMA) technique was developed for structural health monitoring. Liang et al. [5] first proposed a one-dimensional model based on the EMA technique, theoretically analyzing the relationship between the piezoelectric patch and the structural mechanical impedance. They demonstrated that structural damage, by altering the mechanical impedance, could be reflected through changes in electrical admittance, thereby establishing the theoretical foundation for EMA-based techniques in damage detection. Subsequently, Zhou et al. [6] extended the one-dimensional model to a two-dimensional formulation for damage detection in a thin plate. Bhalla and Soh [7] introduced the concept of effective mechanical impedance and derived a coupled electromechanical impedance formula for the piezoelectric sensor-driven system. Additionally, Annamdas and Soh [8,9] proposed a new three-dimensional model based on piezoelectric sensors, incorporating the influence of multiple sensor masses. This more generalized model allows for optimized sensor placement to accurately identify damage locations. Yan et al. [10] employed the method of reverberation ray matrix to analytically investigate the EMA model, clarifying the influence of various physical parameters in the analytical expressions on the electromechanical impedance. These relatively early studies successfully established the theoretical framework of EMA modeling and applied the findings to practical engineering analyses. As the structural complexity continues to increase and the diversity of damage scenarios expands, numerical techniques, especially the finite element method (FEM), have been incorporated into damage detection based on the electromechanical admittance (EMA). Subsequently, the FEM has been demonstrated to be highly efficient in simulating the EMA behavior, yielding results that exhibit a high degree of consistency with experimental data [11,12].

Building upon these theoretical foundations, the EMA technique has been widely adopted for damage detection across diverse engineering structures. For instance, Sapidis et al. [13] developed a novel approach using a one-dimensional convolutional neural network to identify crack initiation based on the raw EMA signatures acquired from externally bonded PZT transducers. Singh et al. [14] introduced a new method based on a uniform sensor network, utilizing EMA-based damage indices to localize multiple damages in glass fiber–reinforced polymer and 3D-printed acrylonitrile butadiene styrene plates. Zhu et al. [15] investigated the temperature effect on the EMA spectra of 2-2 cement-based piezoelectric transducers through combined theoretical and experimental analysis. For the detection of unknown stress and associated damage in concrete structures, Ai and Mo [16] employed a hybrid framework coupling a three-dimensional convolutional neural network with the EMA technique. To overcome the computational inefficiency of the traditional finite element method, Yang et al. [17] proposed an integrated approach combining a meso-element equivalent method with a polynomial spectral element method to simulate EMA-based identification of internal microcracks in concrete structures. Collectively, these studies demonstrated that the drive-point electromechanical admittance (EMA) method offers distinct advantages over conventional non-destructive testing methods. A key factor is its use of high-frequency excitation, which provides two principal benefits. First, in dynamic harmonic response analysis, the short wavelength yields high sensitivity to incipient or minor damage, allowing for early detection and intervention. Second, high-frequency operation inherently suppresses low-frequency environmental noise, resulting in strong anti-interference capability and stable electrical signals. This combination supports reliable, real-time monitoring of structural integrity.

A well-recognized challenge in structural health monitoring is the precise localization of damages. Piezoelectric transducers exhibit a distinct distance-dependent sensitivity to damage, a characteristic that can be leveraged for localization purposes. However, the conventional drive-point EMA technique relies on a single piezoelectric transducer for signal acquisition. Although this approach can detect the presence of damage, it generally provides limited spatial information for damage localization. The cross-EMA technique [18,19,20], which connects multiple piezoelectric patches in parallel, may improve the ability to localize damage. Meher et al. [18] and Meher and Sunny [19,20] combined the cross-EMA technique with artificial neural networks to successfully identify bolt loosening, multiple damages in beams and stiffened plates, as well as delamination/disbonding in composite lap joints, achieving reliable detection results. These findings further validate the enhanced sensitivity of the cross-EM admittance method for damage identification. By measuring the dynamic interaction between two PZT patches, the technique can sensitively capture alterations in stress wave propagation caused by damage along the sensing path, thereby improving the ability to detect minor and incipient damage. However, a scarcity of analytical cross-EMA models for damage detection exists in the literature.

A primary contribution of this study is the development of an analytical cross-EMA model, based on the transfer matrix method (TMM) [21], for localizing cracks in beams. It should be noted that Meher and Sunny [20] established an approximate analytical cross-EMA model based on Euler–Bernoulli beam theory and the modal superposition method, in which the cross-mechanical impedance was derived from modal structural stiffness. In comparison, this study constructs a TMM-based analytical cross-EMA model for cracked beams, where Timoshenko’s beam theory is adopted to describe flexural deformation, and rod theory is employed to characterize axial motion. Different from the existing approximate model [20], the cross EMA derived in this paper is directly obtained based on segment transfer matrices and electromechanical coupling, without introducing any modal approximation assumptions. The key advantage of TMM lies in its ability to perform dynamic analysis of non-uniform beams (with or without bonded PZT wafers) through straightforward matrix multiplication, thereby establishing a global transfer relationship that connects the mechanical variables at the start and end points. Furthermore, regardless of how many segments the beam is discretized into, the dimension of the overall transfer matrix remains constant. In order to verify the accuracy of the proposed cross-EMA model, the analytical solutions were compared with the finite element results and experimental measurements. The robust validation of model accuracy provides a fundamental basis for subsequent damage localization using cross-EMA signatures. A statistical damage index, the correlation coefficient (CC) [22,23,24], was then employed to quantitatively detect cracks in steel beams using the distributed PZT wafers [25,26,27]. Finally, cross-EMA-based experimental measurements were further performed on five steel beams for crack localization.

## 2. Cross-EMA Model Based on TMM

### 2.1. Analytical Formulations

**Figure 1:**
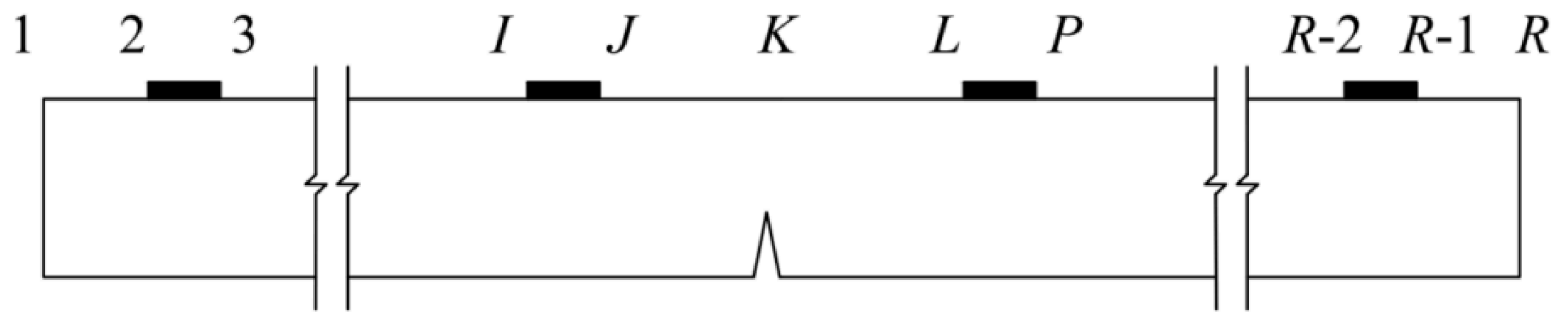
Schematic diagram of a cracked beam.

As shown in Figure 1, multiple piezoelectric wafers are surface-bonded to a cracked beam, serving as actuators or sensors for crack localization. The flexural vibration is modeled using Timoshenko’s beam theory, while axial deformation is described by the classical longitudinal rod assumption. The governing equations for a beam segment without a bonded piezoelectric transducer are thus given as follows:

(1) $$\begin{array}{c} \frac{\partial M}{\partial x}-Q+\rho_{s}I\frac{\partial^{2}\varphi }{\partial t^{2}}=0,\;M=-E_{s}I\frac{\partial \varphi }{\partial x},\;\frac{\partial Q}{\partial x}=\rho_{s}A\frac{\partial^{2}w}{\partial t^{2}},\;\\ Q=\mathrm{\kappa AG}\left(\frac{\partial w}{\partial x}-\varphi \right),\;\frac{\mathrm{dN}}{\mathrm{dx}}=\rho_{s}A\frac{\partial^{2}u}{\partial t^{2}},\;N=E_{s}A\frac{\partial u}{\partial x}. \end{array}$$

The steady-state solution to a harmonic force can be expressed as

(2) $$\begin{array}{l} u=\left(C_{1}e^{ik_{s}x}+C_{2}e^{-ik_{s}x}\right)e^{i\mathrm{\omega t}}\\ w(x,t)=\left(C_{3}e^{k_{1}x}+C_{4}e^{k_{2}x}+C_{5}e^{-k_{1}x}+C_{6}e^{-k_{2}x}\right)e^{i\mathrm{\omega t}},\\ \varphi (x,t)=\left(C_{3}\gamma_{s1}e^{k_{1}x}+C_{4}\gamma_{s2}e^{k_{2}x}-C_{5}\gamma_{s1}e^{-k_{1}x}-C_{6}\gamma_{s2}e^{-k_{2}x}\right)e^{i\mathrm{\omega t}}, \end{array}$$

in which

(3) $$\begin{array}{c} k_{1}=\left\{\begin{array}{c} \sqrt{\frac{-\left(\frac{\omega^{2}}{\kappa c_{s}^{2}}+\frac{\omega^{2}}{c_{0}^{2}}\right)+\sqrt{{\left(\frac{\omega^{2}}{\kappa c_{s}^{2}}-\frac{\omega^{2}}{c_{0}^{2}}\right)}^{2}+\frac{4\omega^{2}}{c_{0}^{2}r_{s}^{2}}}}{2}},\;\mathrm{if}\;\omega <\sqrt{\kappa }c_{s}/r_{s}\\ \sqrt{\frac{\left(\frac{\omega^{2}}{\kappa c_{s}^{2}}+\frac{\omega^{2}}{c_{0}^{2}}\right)-\sqrt{{\left(\frac{\omega^{2}}{\kappa c_{s}^{2}}-\frac{\omega^{2}}{c_{0}^{2}}\right)}^{2}+\frac{4\omega^{2}}{c_{0}^{2}r_{s}^{2}}}}{2}}\cdot i,\;\mathrm{if}\;\omega >\sqrt{\kappa }c_{s}/r \end{array}\right.\\ k_{2}=\sqrt{\frac{\left(\frac{\omega^{2}}{\kappa c_{s}^{2}}+\frac{\omega^{2}}{c_{0}^{2}}\right)+\sqrt{{\left(\frac{\omega^{2}}{\kappa c_{s}^{2}}-\frac{\omega^{2}}{c_{0}^{2}}\right)}^{2}+\frac{4\omega^{2}}{c_{0}^{2}r_{s}^{2}}}}{2}}\cdot i,\\ k_{s}^{2}=\rho_{s}\omega^{2}/E_{s},\;\gamma_{\mathrm{si}}=k_{i}+\frac{\omega^{2}}{\kappa c_{s}^{2}k_{i}},\;(i=1,2). \end{array}$$

where $c_{s}=\sqrt{G/\rho_{s}}$; $c_{0}=\sqrt{E_{s}/\rho_{s}}$; $r_{s}=\sqrt{I/A}=h_{s}/\sqrt{12}$; and *C_i_* (*i* = 1, 2, 3, 4, 5, 6) are undetermined constants.

**Figure 2:**
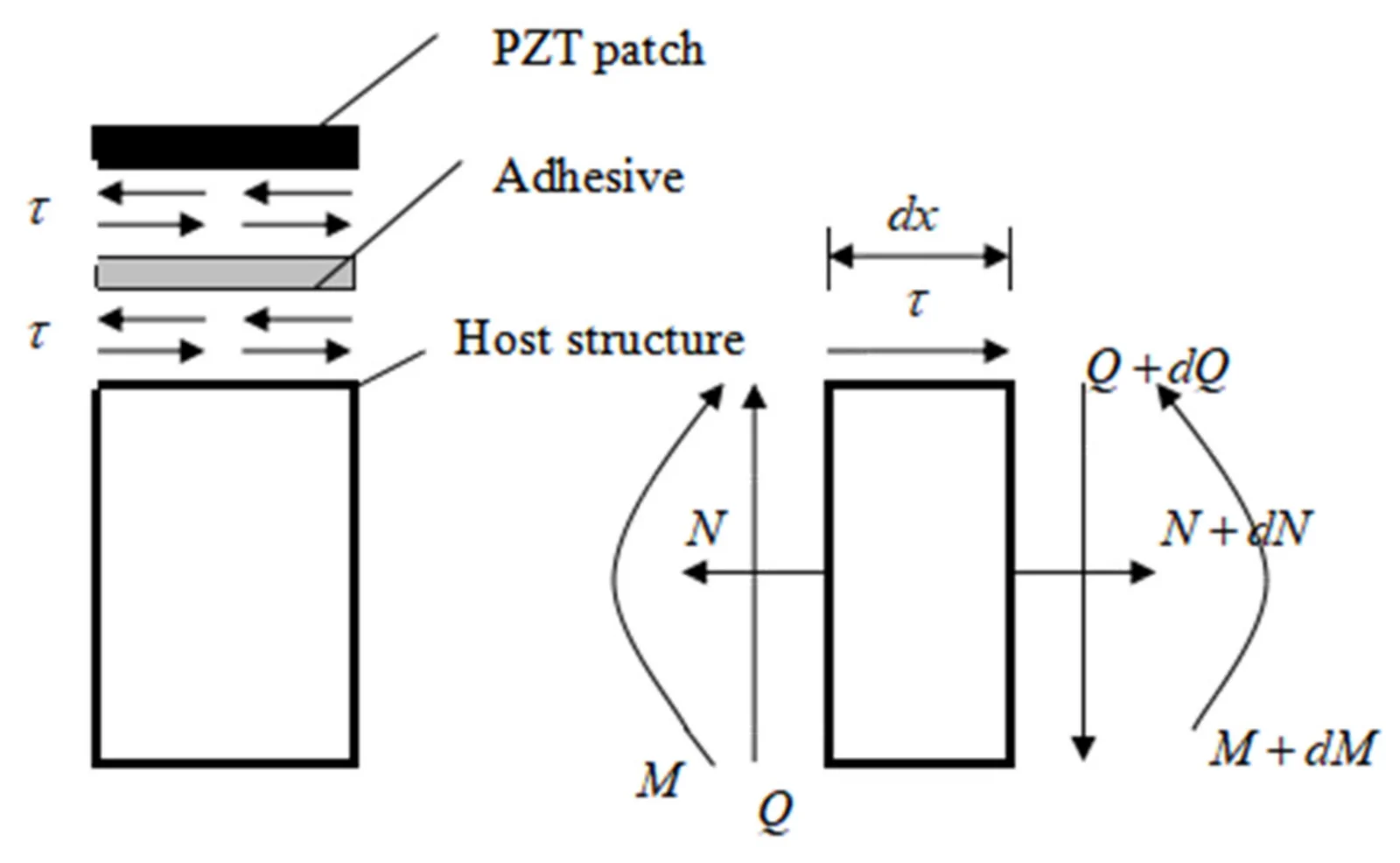
A beam segment with surface-bonded PZT wafer.

For a beam segment integrated with a piezoelectric wafer, as illustrated in Figure 2, the PZT patch is modeled under a 1D axial vibration assumption, whereas the bonding layer is described by the shear lag model [28,29]. Accordingly, the following governing relations are derived:

(4) $$\begin{array}{c} \mathrm{PZT}\;\mathrm{wafer}:\;E_{p}\frac{\partial^{2}u_{p}}{\partial x^{2}}-\frac{\tau (x)}{h_{p}}=\rho_{p}\frac{\partial^{2}u_{p}}{\partial t^{2}},\\ \mathrm{Bonding}\;\mathrm{layer}:\;\tau =\frac{G_{a}}{h_{a}}(u_{p}-u_{s}),\;u_{s}=u-\frac{h_{s}}{2}\frac{\partial w}{\partial x}\\ \mathrm{Host}\mathrm{beam}\mathrm{member}:\;\\ \frac{\partial M}{\partial x}-\tau (x)h_{s}/2-Q+\rho_{s}I\frac{\partial^{2}\varphi }{\partial t^{2}}=0,\;M=-E_{s}I\frac{\partial \varphi }{\partial x},\;\frac{\partial Q}{\partial x}=\rho_{s}A\frac{\partial^{2}w}{\partial t^{2}},\\ Q=\mathrm{\kappa AG}(\frac{\partial w}{\partial x}-\varphi ),\;\frac{\partial N}{\partial x}+\tau (x)=\rho_{s}A\frac{\partial^{2}u}{\partial t^{2}},\;N=E_{s}A\frac{\partial u}{\partial x} \end{array}$$

Defining $k_{p}^{2}=\rho_{p}\omega^{2}/E_{p}$, $k_{G}^{2}=\frac{\rho_{s}\omega^{2}}{\mathrm{\kappa G}}$, $u=\bar{u}e^{\mathrm{i\omega t}}$, $u_{p}={\bar{u}}_{p}e^{\mathrm{i\omega t}}$, $\varphi =\bar{\varphi }e^{\mathrm{i\omega t}}$ and $w=\bar{w}e^{\mathrm{i\omega t}}$ yields the following four differential equations under a harmonic excitation:

(5) $$\begin{array}{c} \bar{u}=\left[1-\frac{(\psi +1)k_{p}^{2}}{\psi {\left(\Gamma /a\right)}^{2}}\right]{\bar{u}}_{p}+\frac{h_{s}}{2}\frac{d\bar{w}}{dx}-\frac{\psi +1}{\psi {\left(\Gamma /a\right)}^{2}}\frac{d^{2}{\bar{u}}_{p}}{dx^{2}},\\ \frac{d\bar{\varphi }}{dx}=\frac{d^{2}\bar{w}}{dx^{2}}+k_{G}^{2}\bar{w},\;\frac{d\bar{w}}{dx}=\alpha_{1}\frac{d^{6}{\bar{u}}_{p}}{dx^{6}}+\alpha_{2}\frac{d^{4}{\bar{u}}_{p}}{dx^{4}}+\alpha_{3}\frac{d^{2}{\bar{u}}_{p}}{dx^{2}}+\alpha_{4}{\bar{u}}_{p},\\ \frac{d^{8}{\bar{u}}_{p}}{dx^{8}}+D_{1}\frac{d^{6}{\bar{u}}_{p}}{dx^{6}}+D_{2}\frac{d^{4}{\bar{u}}_{p}}{dx^{4}}+D_{3}\frac{d^{2}{\bar{u}}_{p}}{dx^{2}}+D_{4}{\bar{u}}_{p}=0, \end{array}$$

where

(6) $$\begin{array}{c} \alpha_{1}=\frac{h_{s}}{6k_{s}^{2}}\frac{\psi +1}{\psi {\left(\Gamma /a\right)}^{2}},\alpha_{2}=-\frac{2h_{s}}{3\psi k_{s}^{2}}-\frac{h_{s}}{6k_{s}^{2}}+\frac{h_{s}}{6k_{s}^{2}}(k_{p}^{2}+k_{s}^{2}+k_{G}^{2})\frac{\psi +1}{\psi {\left(\Gamma /a\right)}^{2}},\\ \alpha_{3}=\frac{h_{s}}{k_{s}^{2}}\left[-\frac{2}{3\psi }k_{p}^{2}-\frac{k_{s}^{2}}{6}-\frac{k_{G}^{2}}{6}-\frac{k_{G}^{2}}{6\psi }+\left(\frac{k_{G}^{2}k_{p}^{2}}{6}+\frac{k_{G}^{2}k_{s}^{2}}{6}+\frac{k_{s}^{2}k_{p}^{2}}{6}\right)\frac{\psi +1}{\psi {\left(\Gamma /a\right)}^{2}}\right],\\ \alpha_{4}=-\frac{h_{s}k_{G}^{2}}{6k_{s}^{2}}\left[k_{p}^{2}/\psi +k_{s}^{2}-\frac{(\psi +1)k_{s}^{2}k_{p}^{2}}{\psi {\left(\Gamma /a\right)}^{2}}\right], \end{array}$$

and

(7) $$\begin{array}{cc} D_{1}= & -\frac{4}{\psi +1}(\Gamma /a{)}^{2}-\frac{\psi }{\psi +1}(\Gamma /a{)}^{2}+k_{p}^{2}+k_{G}^{2}+2k_{s}^{2},\\ D_{2}= & k_{s}^{4}+2k_{s}^{2}k_{p}^{2}+2k_{s}^{2}k_{G}^{2}+k_{G}^{2}k_{p}^{2}-\frac{12k_{s}^{2}}{h_{s}^{2}}-(4k_{s}^{2}+4k_{p}^{2}+k_{G}^{2})\frac{1}{\psi +1}(\Gamma /a{)}^{2}\\ & -(2k_{s}^{2}+k_{G}^{2})\frac{\psi }{\psi +1}(\Gamma /a{)}^{2},\\ D_{3}= & -\left(k_{G}^{2}k_{p}^{2}+4k_{s}^{2}k_{p}^{2}+k_{s}^{2}k_{G}^{2}-\frac{12k_{s}^{2}}{h_{s}^{2}}\right)\frac{1}{\psi +1}(\Gamma /a{)}^{2}\\ & -\left(k_{s}^{4}+2k_{s}^{2}k_{G}^{2}-\frac{12k_{s}^{2}}{h_{s}^{2}}\right)\frac{\psi }{\psi +1}(\Gamma /a{)}^{2}+2k_{G}^{2}k_{p}^{2}k_{s}^{2}+k_{s}^{4}k_{p}^{2}+k_{s}^{4}k_{G}^{2}-\frac{12k_{s}^{2}}{h_{s}^{2}}k_{p}^{2}-\frac{12k_{s}^{4}}{h_{s}^{2}},\\ D_{4}= & \left(k_{s}^{2}k_{G}^{2}-\frac{12k_{s}^{2}}{h_{s}^{2}}\right)\left(-k_{p}^{2}\frac{1}{\psi +1}(\Gamma /a{)}^{2}-k_{s}^{2}\frac{\psi }{\psi +1}{\left(\Gamma /a\right)}^{2}+k_{s}^{2}k_{p}^{2}\right). \end{array}$$

As derived from Equation (5),

(8) $$\begin{array}{c} u_{p}=\Omega Ce^{i\omega t},\;\varphi =\Omega \left[\begin{array}{cc} \mathrm{diag}\left[\lambda_{i}\right] & 0\\ 0 & \mathrm{diag}\left[\lambda_{i}\right] \end{array}\right]Ce^{i\omega t},\\ w=\Omega \left[\begin{array}{cc} \mathrm{diag}\left[\gamma_{i}\right] & 0\\ 0 & \mathrm{diag}\left[-\gamma_{i}\right] \end{array}\right]Ce^{i\omega t},u=\Omega \left[\begin{array}{cc} \mathrm{diag}\left[\eta_{i}\right] & 0\\ 0 & \mathrm{diag}\left[\eta_{i}\right] \end{array}\right]Ce^{i\omega t}, \end{array}$$

where

(9) $$\begin{array}{c} C={\left[\begin{array}{cccccccc} C_{1} & C_{2} & C_{3} & C_{4} & C_{5} & C_{6} & C_{7} & C_{8} \end{array}\right]}^{T},\\ \Omega =\left[\begin{array}{cccccccc} e^{\beta_{1}x} & e^{\beta_{2}x} & e^{\beta_{3}x} & e^{\beta_{4}x} & e^{-\beta_{1}x} & e^{-\beta_{2}x} & e^{-\beta_{3}x} & e^{-\beta_{4}x} \end{array}\right],\\ \gamma_{i}=\alpha_{1}\beta_{i}^{5}+\alpha_{2}\beta_{i}^{3}+\alpha_{3}\beta_{i}+\alpha_{4}/\beta_{i},\;\lambda_{i}=\gamma_{i}\beta_{i}+\gamma_{i}k_{G}^{2}/\beta_{i}\\ \eta_{i}=1-\frac{(\psi +1)k_{p}^{2}}{\psi {\left(\Gamma /a\right)}^{2}}-\frac{\left(\psi +1\right)}{\psi {\left(\Gamma /a\right)}^{2}}\beta_{i}^{2}+\frac{h_{s}}{2}\gamma_{i}\beta_{i},\;(i=1,2,3,4) \end{array}$$

where **diag**[⋅] denotes a diagonal matrix; **C** is an undetermined constant vector; and *β_i_* is the characteristic root of the final equation in Equation (5).

### 2.2. Transfer Matrix Method

In the transfer matrix method (TMM), a key step is to formulate the transfer matrix that relates the physical variables at the two ends of each beam segment. Three typical transfer relationships need to be considered: beam segments without piezoelectric patches; beam segments with bonded patches; and crack location. First, we investigate the beam segment without a piezoelectric patch, taking the *JK* beam element as an example (see Figure 1). From Equations (1) and (2), we obtain

(10) $$V_{J}=M_{J}\bar{C},V_{K}=M_{K}\bar{C},$$

where $V={\left\{\begin{array}{cccccc} u & w & \varphi & N & Q & M \end{array}\right\}}^{T}$; **V***_J_* and **V***_K_* are physical variable vectors of the left and right ends of the *JK* beam segment, respectively; and $\bar{C}={\left\{\begin{array}{cccccc} C_{1} & C_{2} & C_{3} & C_{4} & C_{5} & C_{6} \end{array}\right\}}^{T}$ is an undetermined constant vector. **M***_J_* and **M***_K_* can be directly derived from Equations (1) and (2) as follows:

(11) $$\begin{array}{c} M_{J}=\left[\begin{array}{cccccc} 1 & 1 & 0 & 0 & 0 & 0\\ 0 & 0 & 1 & 1 & 1 & 1\\ 0 & 0 & \gamma_{s1} & \gamma_{s2} & -\gamma_{s1} & -\gamma_{s2}\\ ik_{s}E_{s}A & -ik_{s}E_{s}A & 0 & 0 & 0 & 0\\ 0 & 0 & \mathrm{\kappa AG}(k_{1}-\gamma_{s1}) & \mathrm{\kappa AG}(k_{2}-\gamma_{s2}) & -\mathrm{\kappa AG}(k_{1}-\gamma_{s1}) & -\mathrm{\kappa AG}(k_{2}-\gamma_{s2})\\ 0 & 0 & -E_{s}Ik_{1}\gamma_{s1} & -E_{s}Ik_{2}\gamma_{s2} & -E_{s}Ik_{1}\gamma_{s1} & -E_{s}Ik_{2}\gamma_{s2} \end{array}\right],\\ \begin{array}{l} M_{K}=\left[\begin{array}{ccc} e^{ik_{s}l_{\mathrm{JK}}} & e^{-ik_{s}l_{\mathrm{JK}}} & 0\\ 0 & 0 & e^{k_{1}l_{\mathrm{JK}}}\\ 0 & 0 & \gamma_{s1}e^{k_{1}l_{\mathrm{JK}}}\\ ik_{s}E_{s}Ae^{ik_{s}l_{\mathrm{JK}}} & -ik_{s}E_{s}Ae^{-ik_{s}l_{\mathrm{JK}}} & 0\\ 0 & 0 & \mathrm{\kappa AG}(k_{1}-\gamma_{s1})e^{k_{1}l_{\mathrm{JK}}}\\ 0 & 0 & -E_{s}Ik_{1}\gamma_{s1}e^{k_{1}l_{\mathrm{JK}}} \end{array}\right.\\ \left.\begin{array}{ccc} 0 & 0 & 0\\ e^{k_{2}l_{\mathrm{JK}}} & e^{-k_{1}l_{\mathrm{JK}}} & e^{-k_{2}l_{\mathrm{JK}}}\\ \gamma_{s2}e^{k_{2}l_{\mathrm{JK}}} & -\gamma_{s1}e^{-k_{1}l_{\mathrm{JK}}} & -\gamma_{s2}e^{-k_{2}l_{\mathrm{JK}}}\\ 0 & 0 & 0\\ \mathrm{\kappa AG}(k_{2}-\gamma_{s2})\gamma_{s2}e^{k_{2}l_{\mathrm{JK}}} & -\mathrm{\kappa AG}(k_{1}-\gamma_{s1})e^{-k_{1}l_{\mathrm{JK}}} & -\mathrm{\kappa AG}(k_{2}-\gamma_{s2})e^{-k_{2}l_{\mathrm{JK}}}\\ -E_{s}Ik_{2}\gamma_{s2}e^{k_{2}l_{\mathrm{JK}}} & -E_{s}Ik_{1}\gamma_{s1}e^{-k_{1}l_{\mathrm{JK}}} & -E_{s}Ik_{2}\gamma_{s2}e^{-k_{2}l_{\mathrm{JK}}} \end{array}\right]. \end{array} \end{array}$$

Equation (10) yields

(12) $$V_{K}=T_{KJ}V_{J},\;\mathrm{and}\;T_{KJ}=M_{K}M_{J}^{-1}.$$

where **T***_KJ_* is the transfer matrix of order 6 × 6, which establishes the transfer relation between physical variables of the left side and right side of the *JK* beam member. Similarly, the transfer relations for all beam segments without bonded PZT patches can be derived. In addition, at crack point *K*, we can obtain the following relations [30]:

(13) $$\begin{array}{c} u_{K}^{-}=u_{K}^{+},\;N_{K}^{-}=N_{K}^{+},\;w_{K}^{-}=w_{K}^{+},\;M_{K}^{-}=M_{K}^{+},\;Q_{K}^{-}=Q_{K}^{+},\\ {\left(\frac{\partial w}{\partial x}\right)}_{K}^{+}-{\left(\frac{\partial w}{\partial x}\right)}_{K}^{-}=\mathrm{\theta l}{\left(\frac{\partial \varphi }{\partial x}\right)}_{K}^{+}. \end{array}$$

The equivalence of shear forces on both sides of the crack, by combining the last expression in Equation (13) with Equation (1), yields:

(14) $$\varphi_{K}^{+}-\varphi_{K}^{-}={\left(\frac{\partial w}{\partial x}\right)}_{K}^{+}-{\left(\frac{\partial w}{\partial x}\right)}_{K}^{-}=\mathrm{\theta l}{\left(\frac{\partial \varphi }{\partial x}\right)}_{K}^{+}$$

where superscripts “ − ” and “ + ” denote the left side and right side of the crack, respectively. For a single-sided open crack, we have [30]:

(15) $$\begin{array}{c} \theta =6\pi \gamma^{2}f_{J}(\gamma )\left(\frac{h_{s}}{l}\right),\;\gamma =\frac{c}{h_{s}}\\ f_{J}(\gamma )=0.6384-1.035\gamma +3.7201\gamma^{2}-5.1773\gamma^{3}+7.553\gamma^{4}-7.332\gamma^{5}+2.4909\gamma^{6}. \end{array}$$

The expressions in Equations (13) and (14) lead to the following transfer relation between the left and right sides of the crack:

(16) $$V_{k^{+}}=T_{k^{+}k^{-}}V_{k^{-}},$$

where $T_{k^{+}k^{-}}=M_{k^{+}}M_{k^{-}}^{-1}$, and

(17) $$\begin{array}{c} M_{K^{-}}=\left[\begin{array}{cccccc} 1 & 1 & 0 & 0 & 0 & 0\\ 0 & 0 & 1 & 1 & 1 & 1\\ 0 & 0 & \gamma_{s1} & \gamma_{s2} & -\gamma_{s1} & -\gamma_{s2}\\ ik_{s}E_{s}A & -ik_{s}E_{s}A & 0 & 0 & 0 & 0\\ 0 & 0 & \mathrm{\kappa AG}(k_{1}-\gamma_{s1}) & \mathrm{\kappa AG}(k_{2}-\gamma_{s2}) & -\mathrm{\kappa AG}(k_{1}-\gamma_{s1}) & -\mathrm{\kappa AG}(k_{2}-\gamma_{s2})\\ 0 & 0 & -E_{s}Ik_{1}\gamma_{s1} & -E_{s}Ik_{2}\gamma_{s2} & -E_{s}Ik_{1}\gamma_{s1} & -E_{s}Ik_{2}\gamma_{s2} \end{array}\right],\\ M_{K^{+}}=\left[\begin{array}{cccccc} 1 & 1 & 0 & 0 & 0 & 0\\ 0 & 0 & 1 & 1 & 1 & 1\\ 0 & 0 & \gamma_{s1}-\mathrm{\theta l}k_{1}\gamma_{s1} & \gamma_{s2}-\mathrm{\theta l}k_{2}\gamma_{s2} & -\gamma_{s1}-\mathrm{\theta l}k_{1}\gamma_{s1} & -\gamma_{s2}-\mathrm{\theta l}k_{2}\gamma_{s2}\\ ik_{s}E_{s}A & -ik_{s}E_{s}A & 0 & 0 & 0 & 0\\ 0 & 0 & \mathrm{\kappa AG}(k_{1}-\gamma_{s1}) & \mathrm{\kappa AG}(k_{2}-\gamma_{s2}) & -\mathrm{\kappa AG}(k_{1}-\gamma_{s1}) & -\mathrm{\kappa AG}(k_{2}-\gamma_{s2})\\ 0 & 0 & -E_{s}Ik_{1}\gamma_{s1} & -E_{s}Ik_{2}\gamma_{s2} & -E_{s}Ik_{1}\gamma_{s1} & -E_{s}Ik_{2}\gamma_{s2} \end{array}\right]. \end{array}$$

For a beam element integrated with a piezoelectric wafer (e.g., the *LP* segment), the system is described by eight unknown constants in Equations (4) and (8), whereas the physical state of the beam is defined by only six variables. Therefore, it is challenging to directly establish a local transfer relation for this beam segment. The resolution lies in introducing the boundary conditions at the two ends of the PZT patch [28,29], which are provided below:

(18) $$\varepsilon_{p}=\frac{\partial u_{p}}{\partial x}=d_{31}E_{3}=d_{31}V/h_{p}=\Lambda .$$

Thus, we can obtain

(19) $$\begin{array}{c} {\left\{\begin{array}{cccccccc} u^{L} & w^{L} & \varphi^{L} & N^{L} & Q^{L} & M^{L} & \Lambda & \Lambda \end{array}\right\}}^{T}={\bar{M}}_{L}C,\\ {\left\{\begin{array}{cccccccc} u^{P} & w^{P} & \varphi^{P} & N^{P} & Q^{P} & M^{P} & u_{p}^{L} & u_{p}^{P} \end{array}\right\}}^{T}={\bar{M}}_{P}C, \end{array}$$

where

(20) $$\begin{array}{c} \begin{array}{l} {\bar{M}}_{L}=\left[\begin{array}{cccc} \eta_{1} & \eta_{2} & \eta_{3} & \eta_{4}\\ \gamma_{1} & \gamma_{2} & \gamma_{3} & \gamma_{4}\\ \lambda_{1} & \lambda_{2} & \lambda_{3} & \lambda_{4}\\ E_{s}A\eta_{1}\beta_{1} & E_{s}A\eta_{2}\beta_{2} & E_{s}A\eta_{3}\beta_{3} & E_{s}A\eta_{4}\beta_{4}\\ \mathrm{\kappa AG}(\gamma_{1}\beta_{1}-\lambda_{1}) & \mathrm{\kappa AG}(\gamma_{2}\beta_{2}-\lambda_{2}) & \mathrm{\kappa AG}(\gamma_{3}\beta_{3}-\lambda_{3}) & \mathrm{\kappa AG}(\gamma_{4}\beta_{4}-\lambda_{4})\\ -E_{s}I\beta_{1}\lambda_{1} & -E_{s}I\beta_{2}\lambda_{2} & -E_{s}I\beta_{3}\lambda_{3} & -E_{s}I\beta_{4}\lambda_{4}\\ \beta_{1} & \beta_{2} & \beta_{3} & \beta_{4}\\ \beta_{1}e^{\beta_{1}l_{p}} & \beta_{2}e^{\beta_{2}l_{p}} & \beta_{3}e^{\beta_{3}l_{p}} & \beta_{4}e^{\beta_{4}l_{p}} \end{array}\right.\\ \left.\begin{array}{cccc} \eta_{1} & \eta_{2} & \eta_{3} & \eta_{4}\\ -\gamma_{1} & -\gamma_{2} & -\gamma_{3} & -\gamma_{4}\\ \lambda_{1} & \lambda_{2} & \lambda_{3} & \lambda_{4}\\ -E_{s}A\eta_{1}\beta_{1} & -E_{s}A\eta_{2}\beta_{2} & -E_{s}A\eta_{3}\beta_{3} & -E_{s}A\eta_{4}\beta_{4}\\ \mathrm{\kappa AG}(\gamma_{1}\beta_{1}-\lambda_{1}) & \mathrm{\kappa AG}(\gamma_{2}\beta_{2}-\lambda_{2}) & \mathrm{\kappa AG}(\gamma_{3}\beta_{3}-\lambda_{3}) & \mathrm{\kappa AG}(\gamma_{4}\beta_{4}-\lambda_{4})\\ E_{s}I\beta_{1}\lambda_{1} & E_{s}I\beta_{2}\lambda_{2} & E_{s}I\beta_{3}\lambda_{3} & E_{s}I\beta_{4}\lambda_{4}\\ -\beta_{1} & -\beta_{2} & -\beta_{3} & -\beta_{4}\\ -\beta_{1}e^{-\beta_{1}l_{p}} & -\beta_{2}e^{-\beta_{2}l_{p}} & -\beta_{3}e^{-\beta_{3}l_{p}} & -\beta_{4}e^{-\beta_{4}l_{p}} \end{array}\right] \end{array},\\ \begin{array}{l} {\bar{M}}_{P}=\left[\begin{array}{cccc} \eta_{1}e^{\beta_{1}l_{p}} & \eta_{2}e^{\beta_{2}l_{p}} & \eta_{3}e^{\beta_{3}l_{p}} & \eta_{4}e^{\beta_{4}l_{p}}\\ \gamma_{1}e^{\beta_{1}l_{p}} & \gamma_{2}e^{\beta_{2}l_{p}} & \gamma_{3}e^{\beta_{3}l_{p}} & \gamma_{4}e^{\beta_{4}l_{p}}\\ \lambda_{1}e^{\beta_{1}l_{p}} & \lambda_{2}e^{\beta_{2}l_{p}} & \lambda_{3}e^{\beta_{3}l_{p}} & \lambda_{4}e^{\beta_{4}l_{p}}\\ E_{s}A\eta_{1}\beta_{1}e^{\beta_{1}l_{p}} & E_{s}A\eta_{2}\beta_{2}e^{\beta_{2}l_{p}} & E_{s}A\eta_{3}\beta_{3}e^{\beta_{3}l_{p}} & E_{s}A\eta_{4}\beta_{4}e^{\beta_{4}l_{p}}\\ \mathrm{\kappa AG}(\gamma_{1}\beta_{1}-\lambda_{1})e^{\beta_{1}l_{p}} & \mathrm{\kappa AG}(\gamma_{2}\beta_{2}-\lambda_{2})e^{\beta_{2}l_{p}} & \mathrm{\kappa AG}(\gamma_{3}\beta_{3}-\lambda_{3})e^{\beta_{3}l_{p}} & \mathrm{\kappa AG}(\gamma_{4}\beta_{4}-\lambda_{4})e^{\beta_{4}l_{p}}\\ -E_{s}I\beta_{1}\lambda_{1}e^{\beta_{1}l_{p}} & -E_{s}I\beta_{2}\lambda_{2}e^{\beta_{2}l_{p}} & -E_{s}I\beta_{3}\lambda_{3}e^{\beta_{3}l_{p}} & -E_{s}I\beta_{4}\lambda_{4}e^{\beta_{4}l_{p}}\\ 1 & 1 & 1 & 1\\ e^{\beta_{1}l_{p}} & e^{\beta_{2}l_{p}} & e^{\beta_{3}l_{p}} & e^{\beta_{4}l_{p}} \end{array}\right.\\ \left.\begin{array}{cccc} \eta_{1}e^{-\beta_{1}l_{p}} & \eta_{2}e^{-\beta_{2}l_{p}} & \eta_{3}e^{-\beta_{3}l_{p}} & \eta_{4}e^{-\beta_{4}l_{p}}\\ -\gamma_{1}e^{-\beta_{1}l_{p}} & -\gamma_{2}e^{-\beta_{2}l_{p}} & -\gamma_{3}e^{-\beta_{3}l_{p}} & -\gamma_{4}e^{-\beta_{4}l_{p}}\\ \lambda_{1}e^{-\beta_{1}l_{p}} & \lambda_{2}e^{-\beta_{2}l_{p}} & \lambda_{3}e^{-\beta_{3}l_{p}} & \lambda_{4}e^{-\beta_{4}l_{p}}\\ -E_{s}A\eta_{1}\beta_{1}e^{-\beta_{1}l_{p}} & -E_{s}A\eta_{2}\beta_{2}e^{-\beta_{2}l_{p}} & -E_{s}A\eta_{3}\beta_{3}e^{-\beta_{3}l_{p}} & -E_{s}A\eta_{4}\beta_{4}e^{-\beta_{4}l_{p}}\\ \mathrm{\kappa AG}(\gamma_{1}\beta_{1}-\lambda_{1})e^{-\beta_{1}l_{p}} & \mathrm{\kappa AG}(\gamma_{2}\beta_{2}-\lambda_{2})e^{-\beta_{2}l_{p}} & \mathrm{\kappa AG}(\gamma_{3}\beta_{3}-\lambda_{3})e^{-\beta_{3}l_{p}} & \mathrm{\kappa AG}(\gamma_{4}\beta_{4}-\lambda_{4})e^{-\beta_{4}l_{p}}\\ E_{s}I\beta_{1}\lambda_{1} & E_{s}I\beta_{2}\lambda_{2}e^{-\beta_{2}l_{p}} & E_{s}I\beta_{3}\lambda_{3}e^{-\beta_{3}l_{p}} & E_{s}I\beta_{4}\lambda_{4}e^{-\beta_{4}l_{p}}\\ 1 & 1 & 1 & 1\\ e^{-\beta_{1}l_{p}} & e^{-\beta_{2}l_{p}} & e^{-\beta_{3}l_{p}} & e^{-\beta_{4}l_{p}} \end{array}\right] \end{array}. \end{array}$$

Equation (19) gives

(21) $${\left\{\begin{array}{cccccccc} u^{P} & w^{P} & \varphi^{P} & N^{P} & Q^{P} & M^{P} & u_{p}^{L} & u_{p}^{P} \end{array}\right\}}^{T}={\bar{M}}_{P}{\bar{M}}_{L}^{-1}{\left\{\begin{array}{cccccccc} u^{L} & w^{L} & \varphi^{L} & N^{L} & Q^{L} & M^{L} & \Lambda & \Lambda \end{array}\right\}}^{T}.$$

Setting ${\bar{T}}_{\mathrm{PL}}={\bar{M}}_{P}{\bar{M}}_{L}^{-1}$ yields:

(22) $$V_{P}=T_{PL}V_{L}+Q_{0},$$

where

(23) $$T_{\mathrm{PL}}=\left[\begin{array}{cccccc} {\bar{T}}_{\mathrm{PL}}^{1,1} & {\bar{T}}_{\mathrm{PL}}^{1,2} & {\bar{T}}_{\mathrm{PL}}^{1.3} & {\bar{T}}_{\mathrm{PL}}^{1,4} & {\bar{T}}_{\mathrm{PL}}^{1,5} & {\bar{T}}_{\mathrm{PL}}^{1,6}\\ {\bar{T}}_{\mathrm{PL}}^{2,1} & {\bar{T}}_{\mathrm{PL}}^{2,2} & {\bar{T}}_{\mathrm{PL}}^{2,3} & {\bar{T}}_{\mathrm{PL}}^{2,4} & {\bar{T}}_{\mathrm{PL}}^{2,5} & {\bar{T}}_{\mathrm{PL}}^{2,6}\\ {\bar{T}}_{\mathrm{PL}}^{3,1} & {\bar{T}}_{\mathrm{PL}}^{3,2} & {\bar{T}}_{\mathrm{PL}}^{3,3} & {\bar{T}}_{\mathrm{PL}}^{3,4} & {\bar{T}}_{\mathrm{PL}}^{3,5} & {\bar{T}}_{\mathrm{PL}}^{3,6}\\ {\bar{T}}_{\mathrm{PL}}^{4,1} & {\bar{T}}_{\mathrm{PL}}^{4,2} & {\bar{T}}_{\mathrm{PL}}^{4,3} & {\bar{T}}_{\mathrm{PL}}^{4,4} & {\bar{T}}_{\mathrm{PL}}^{4,5} & {\bar{T}}_{\mathrm{PL}}^{4,6}\\ {\bar{T}}_{\mathrm{PL}}^{5,1} & {\bar{T}}_{\mathrm{PL}}^{5,2} & {\bar{T}}_{\mathrm{PL}}^{5,3} & {\bar{T}}_{\mathrm{PL}}^{5,4} & {\bar{T}}_{\mathrm{PL}}^{5,5} & {\bar{T}}_{\mathrm{PL}}^{5,6}\\ {\bar{T}}_{\mathrm{PL}}^{6,1} & {\bar{T}}_{\mathrm{PL}}^{6,2} & {\bar{T}}_{\mathrm{PL}}^{6,3} & {\bar{T}}_{\mathrm{PL}}^{6,4} & {\bar{T}}_{\mathrm{PL}}^{6,5} & {\bar{T}}_{\mathrm{PL}}^{6,6} \end{array}\right],\;Q_{0}=\left\{\begin{array}{c} {\bar{T}}_{\mathrm{PL}}^{1,7}+{\bar{T}}_{\mathrm{PL}}^{1,8}\\ {\bar{T}}_{\mathrm{PL}}^{2,7}+{\bar{T}}_{\mathrm{PL}}^{2,8}\\ {\bar{T}}_{\mathrm{PL}}^{3,7}+{\bar{T}}_{\mathrm{PL}}^{3,8}\\ {\bar{T}}_{\mathrm{PL}}^{4,7}+{\bar{T}}_{\mathrm{PL}}^{4,8}\\ {\bar{T}}_{\mathrm{PL}}^{5,7}+{\bar{T}}_{\mathrm{PL}}^{5,8}\\ {\bar{T}}_{\mathrm{PL}}^{6,7}+{\bar{T}}_{\mathrm{PL}}^{6,8} \end{array}\right\}\Lambda .$$

where **T***_PL_* is the transfer matrix of order 6 × 6, which establishes the transfer relation between the physical variables of the left side and right side of the *LP* beam member (with a bonded PZT wafer). When a piezoelectric wafer serves only as a sensor and, thus, no voltage is applied across it, we have *Λ* = 0, i.e., **Q**_0_ = **0**. Without loss of generality, applying a harmonic voltage to the PZT patch *LP* (acting as an actuator) enables the derivation of the global transfer relation for the cracked beam, given by:

(24) $$V_{R}={TV}_{1}+Q,Q=T_{R,P}Q_{0}$$

where

(25) $$T=\prod_{m=R-1}^{1}T_{m+1,m},\;T_{R,P}=\prod_{m=R-1}^{P}T_{m+1,m}.$$

Meanwhile, the free-free boundary conditions are always assumed in the EMA-based damage detection technique [31]. Thus, we have

(26) M=0, Q=0, N=0,

at the two sides of the beam. By applying the boundary conditions in conjunction with Equation (24), we derive:

(27) $$\left\{\begin{array}{c} 0\\ 0\\ 0 \end{array}\right\}=\left[\begin{array}{ccc} {Τ}^{4,1} & {Τ}^{4,2} & {Τ}^{4,3}\\ {Τ}^{5,1} & {Τ}^{5,2} & {Τ}^{5,3}\\ {Τ}^{6,1} & {Τ}^{6,2} & {Τ}^{6,3} \end{array}\right]{\left\{\begin{array}{c} u\\ w\\ \varphi \end{array}\right\}}_{1}+\left\{\begin{array}{c} Q^{4,1}\\ Q^{5,1}\\ Q^{6,1} \end{array}\right\}.$$

Accordingly, the displacement at the left end of the beam is given by:

(28) $${\left\{\begin{array}{c} u\\ w\\ \varphi \end{array}\right\}}_{1}=-{\left[\begin{array}{ccc} {Τ}^{4,1} & {Τ}^{4,2} & {Τ}^{4,3}\\ {Τ}^{5,1} & {Τ}^{5,2} & {Τ}^{5,3}\\ {Τ}^{6,1} & {Τ}^{6,2} & {Τ}^{6,3} \end{array}\right]}^{-1}\left\{\begin{array}{c} Q^{4,1}\\ Q^{5,1}\\ Q^{6,1} \end{array}\right\}.$$

where **Q***^i^*^,*j*^ and **Τ***^i^*^,*j*^ represent the corresponding elements of the matrices **Q** and **T**, respectively. Once all the physical variables at the left end of the beam are obtained, the displacement and internal forces at any point along the beam can be determined through the transfer relations. If the physical variables of the beam segment at the left side of a certain piezoelectric wafer are known, the axial displacements at both ends of this PZT wafer can be calculated using Equation (21). Taking the *LP* PZT as an example, we have:

(29) $$\left\{\begin{array}{c} u_{p}^{L}\\ u_{p}^{P} \end{array}\right\}=\left[\begin{array}{cccccc} {\bar{T}}_{\mathrm{PL}}^{7,1} & {\bar{T}}_{\mathrm{PL}}^{7,2} & {\bar{T}}_{\mathrm{PL}}^{7,3} & {\bar{T}}_{\mathrm{PL}}^{7,4} & {\bar{T}}_{\mathrm{PL}}^{7,5} & {\bar{T}}_{\mathrm{PL}}^{7,6}\\ {\bar{T}}_{\mathrm{PL}}^{8,1} & {\bar{T}}_{\mathrm{PL}}^{8,2} & {\bar{T}}_{\mathrm{PL}}^{8,3} & {\bar{T}}_{\mathrm{PL}}^{8,4} & {\bar{T}}_{\mathrm{PL}}^{8,5} & {\bar{T}}_{\mathrm{PL}}^{8,6} \end{array}\right]V_{L}+\left\{\begin{array}{c} {\bar{T}}_{\mathrm{PL}}^{7,7}+{\bar{T}}_{\mathrm{PL}}^{7,8}\\ {\bar{T}}_{\mathrm{PL}}^{8,7}+{\bar{T}}_{\mathrm{PL}}^{8,8} \end{array}\right\}\Lambda .$$

Here, the *LP* PZT acts as both a sensor and an actuator. The other PZT sensors, serving as pure sensors, can be accounted for by setting *Λ* = 0 in Equation (29).

### 2.3. Cross-EMA Model

**Figure 3:**
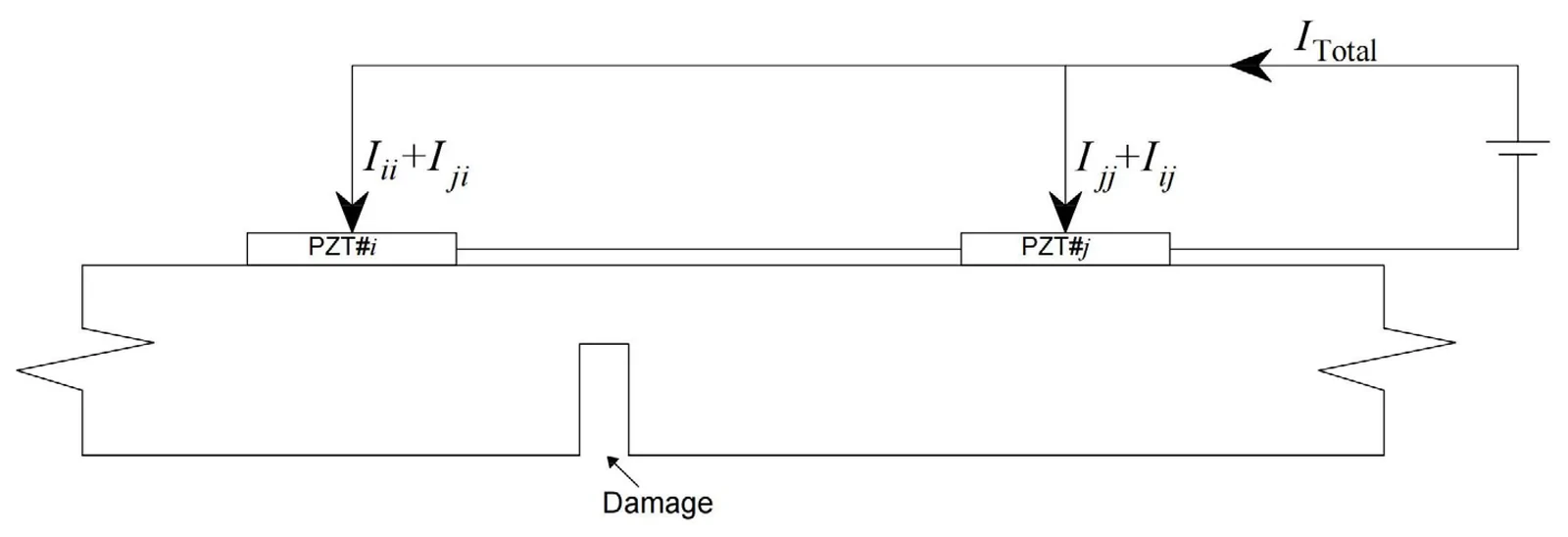
Schematic diagram of cross-EM admittance.

The cross-electromechanical admittance *Y_ij_* between two piezoelectric transducers is theoretically defined as the ratio of the current measured in the *i*-th piezoelectric patch to the voltage applied to the *j*-th piezoelectric patch. Experimentally, two piezoelectric wafers *i* and *j* are connected in parallel and excited by a harmonic voltage, as illustrated in Figure 3. The total electric current through the circuit system is given by [18,19,20]:

(30) $$I_{\mathrm{Total}}=I_{ii}+I_{ij}+I_{ji}+I_{jj},$$

where *I_ij_* represent the electric current at the *i*-th piezoelectric patch when a harmonic voltage excitation is applied to the *j*-th piezoelectric patch. Then, the group EM admittance can be written as follows:

(31) $$Y=\frac{I_{\mathrm{Total}}}{V}=\frac{I_{\mathrm{ii}}+I_{\mathrm{ij}}+I_{\mathrm{ji}}+I_{\mathrm{jj}}}{V}=Y_{\mathrm{ii}}+Y_{\mathrm{ij}}+Y_{\mathrm{ji}}+Y_{\mathrm{jj}}.$$

In Equation (31), *Y_ii_* and *Y_jj_* denote the drive-point electromechanical admittance measured from the *i*-th and *j*-th piezoelectric patches, respectively. Notably, *Y_ij_* and *Y_ji_* are equal for identical parallel piezoelectric transducers within the same group [18,19,20]. Consequently, the cross-EM admittance can be mathematically expressed as:

(32) $$Y_{\mathrm{ij}}=Y_{\mathrm{ji}}=\frac{Y-Y_{\mathrm{ii}}-Y_{\mathrm{jj}}}{2}.$$

In this study, an analytical model will be directly developed according to the definition of cross EMA. As mentioned above, the piezoelectric wafer is taken into account as a thin bar undergoing only axial motion [29,31]. Thus, the constitutive equations [29,31] are as follows:

(33) $$\varepsilon_{p}=\frac{T_{1}}{{\bar{E}}_{p}}+d_{31}E_{3},\;D_{3}={\bar{\varepsilon }}_{33}^{T}E_{3}+d_{31}T_{1},$$

If a unit voltage is applied to the *j*-th PZT actuator, the electric current flowing through the *i*-th PZT sensor is related to the electric displacement as follows [29,31]:

(34) $$I_{i}=i\omega \iint D_{3}\mathrm{dxdy}=i\omega w_{p}l_{p}({\bar{\varepsilon }}_{33}^{T}-d_{31}^{2}{\bar{E}}_{p})E_{3}+i\omega w_{p}d_{31}{\bar{E}}_{p}(u_{p}^{R}-u_{p}^{L})$$

where $u_{p}^{R}$ and $u_{p}^{L}$ denote the axial displacement on the right and left sides of the *i*-th piezoelectric sensor, respectively, and can be determined from Equation (29). According to the definition of cross-EM admittance, we have

(35) $$Y_{\mathrm{ij}}=I_{i}/V_{j}=i\omega w_{p}l_{p}({\bar{\varepsilon }}_{33}^{T}-d_{31}^{2}{\bar{E}}_{p})/h_{p}+i\omega w_{p}d_{31}{\bar{E}}_{p}(u_{p}^{R}-u_{p}^{L})$$

The first term on the right-hand side of Equation (35) is only related to the piezoelectric transducer, and all of the involved parameters are known. The second term incorporates the axial displacement on the right and left sides of the *i*-th piezoelectric sensor, indicating that it reflects the global vibration characteristics of the intelligent beam. It is important to note that when the *j*-th PZT serves as both actuator and sensor, Equation (35) reduces to the expression for *Y_jj_*, thereby defining the conventional drive-point EM admittance. A key distinction is that the cross conductance can take on positive, negative, or zero values, whereas the drive-point conductance is exclusively positive.

## 3. Numerical Results

### 3.1. Comparative Study

**Figure 4:**
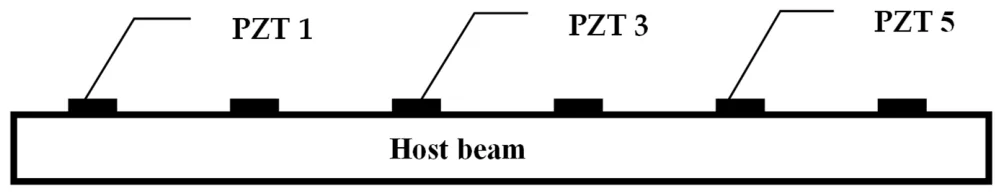
A beam bonded with multiple PZT patches.

To validate the present model, analytical results of electrical admittance were compared with the experimental measurements as well as the finite element method (FEM) simulations. A steel beam specimen with dimensions of 500 mm × 30 mm × 16 mm, a mass density of 7850 kg/m^3^, an elastic modulus of 210 GPa, and a Poisson’s ratio of 0.3 is selected for a comparative study, as depicted in Figure 4. The material coefficients and geometric parameters of the PZT piezoelectric patches are presented in Table 1. In this study, the shear stiffness of the bond layer is always assumed to be 1 GPa [32]. By selecting different adhesive layer thicknesses and substituting them into the expression for the shear lag parameter given in the Nomenclature Section, different shear lag parameters can be obtained. In this case, the adhesive layer thickness is assumed to be 0.03 mm [33].

**Figure 5:**
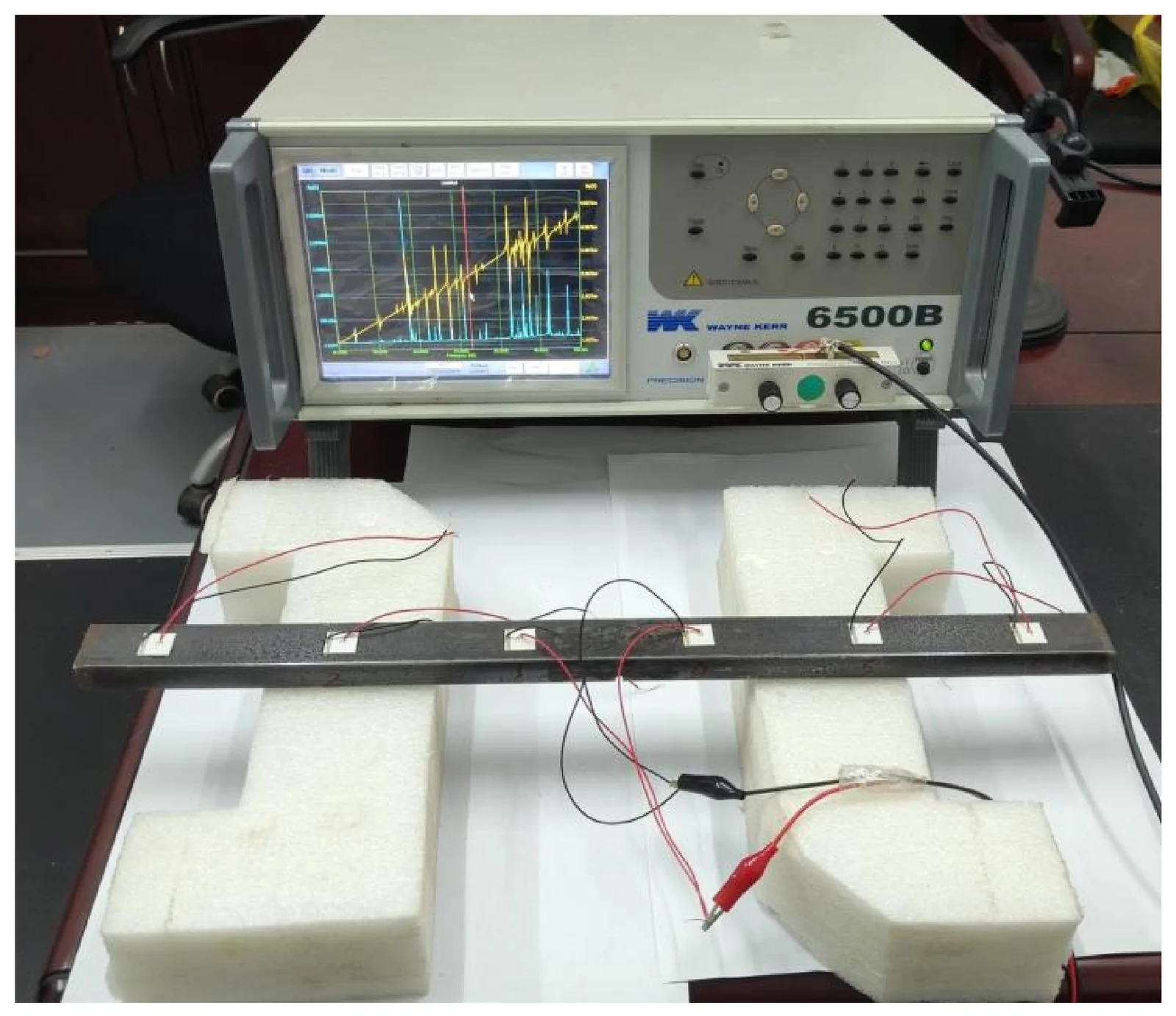
Experimental setup.

The experimental setup employed a precision impedance analyzer (WK-6500B, Wayne Kerr, Wayne Kerr Electronics, Shenzhen, China), as illustrated in Figure 5. Six rectangular piezoelectric transducers were surface-bonded to the steel specimen to act as actuators/sensors. Prior to bonding the PZT patches (Model HNYA16-0.41-1, Suzhou Pante Electronic Ceramics Technologies Co., Ltd., Suzhou, China), the uneven surface of the steel specimen was ground to a smooth finish using sandpaper. The 502 adhesive produced by Ningbo Deli Group Co., Ltd., Ningbo, China, was adopted to bond the PZT patch onto the pre-treated surface of the steel specimen. It is worth noting that the adhesive-bonded transducer requires a 24 h curing period before formal testing, which effectively avoids data drift caused by incomplete curing. Following the complete curing of the 502 adhesive, the lead wires of the PZT patch were connected to the impedance analyzer via alligator clips in the experimental setup. To fully capture the variation trend of the electromechanical admittance, the number of signal acquisition points in each frequency band was set to 800, and the excitation output voltage of the impedance analyzer was configured as 1 V.

Meanwhile, the SOLID226 element with KEYOPT(1) = 1001 was employed to model the piezoelectric materials in the finite element simulation. This 20-node hexahedral element incorporates multiple degrees of freedom per node, making it particularly suitable for analyzing multi-field coupling phenomena in solid structures. In contrast, the beam specimen was modeled via SOLID185 elements, a type of 8-node hexahedral element with three translational degrees of freedom (x, y, and z directions) assigned to each node, which is specifically developed for the modeling of three-dimensional solid continua. To guarantee computational accuracy, the rectangular steel specimen was discretized into hexahedral elements using a mapping meshing strategy. The damping ratio of the steel specimen was set to 0.01 in the model. Subsequently, an alternating voltage with an amplitude of 1 V was applied to the upper and lower surfaces of the PZT patches. The charge values were then extracted within TimeHistPostpro via the ANSYS R19.2 GUI interface. Finally, the electromechanical admittance (EMA) signatures can be obtained from the established finite element model.

**Figure 6:**
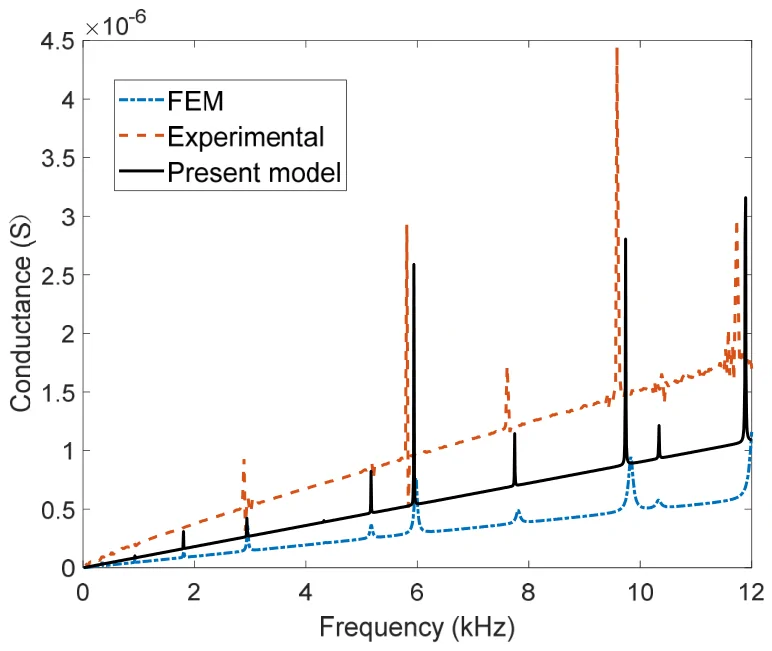
Comparison of drive-point EM conductance for PZT3.

**Figure 7:**
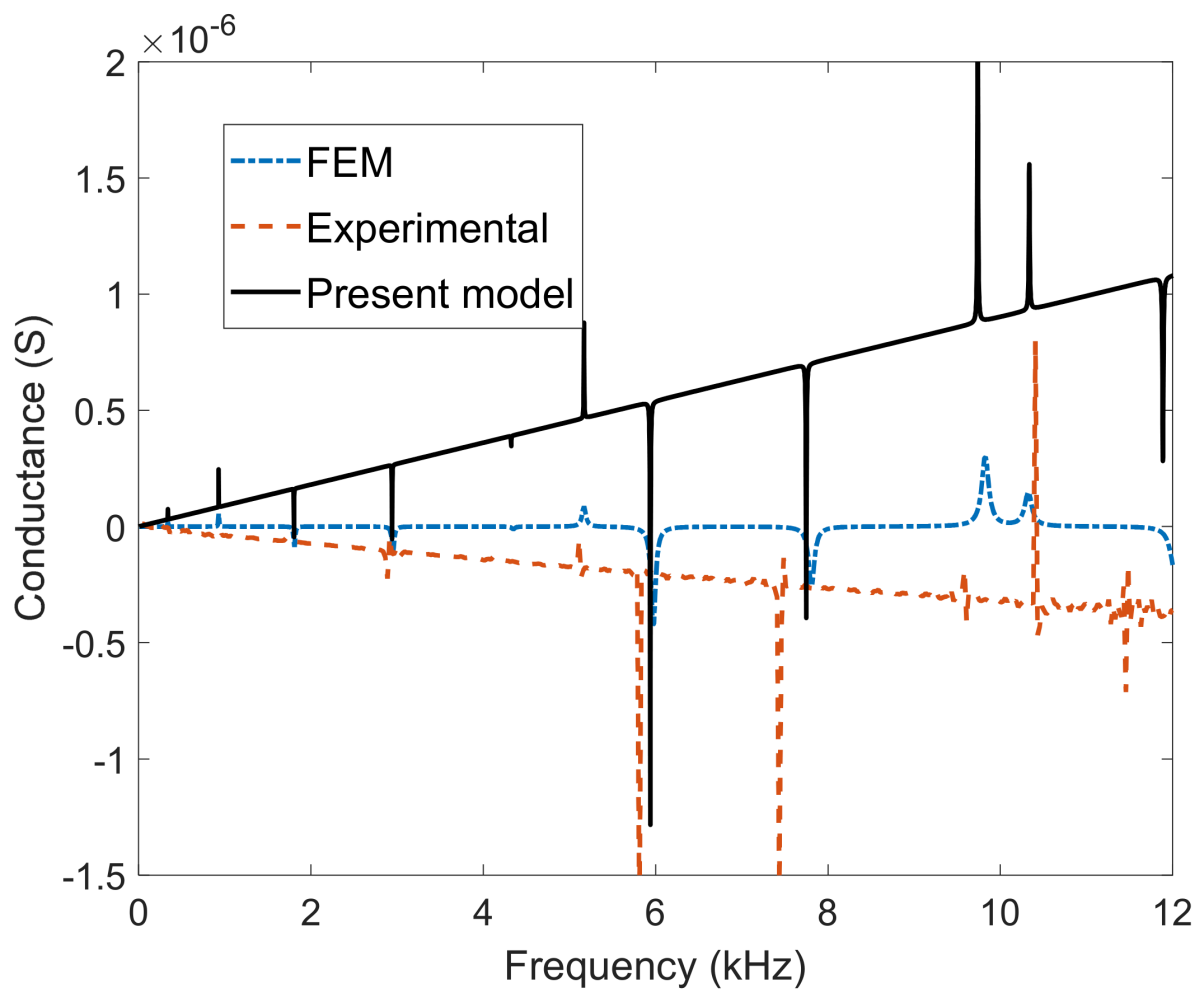
Comparison of cross-EM conductance for PZT2-3.

The drive-point conductance, which represents a particular form of cross conductance, is also investigated for comparison purposes. Figure 6 demonstrates a general consistency in the modal frequencies of the drive-point conductance curve among the experimental, finite element analysis, and simulated results, although some variations exist. The experimental cross-electromechanical (EM) conductance in Figure 7 is not directly measured but derived from Equation (32), which contributes to the relatively larger deviations observed. A similar phenomenon has been reported in Ref. [19]. Nevertheless, the three cross-EMA curves exhibit good agreement in the locations of their peak values. This comparative study confirms the satisfactory accuracy and feasibility of the proposed model for cross-EMA analysis. The following section will further explore damage localization in a steel specimen using this analytical model.

### 3.2. Crack Localization Using the Present Model

**Figure 8:**
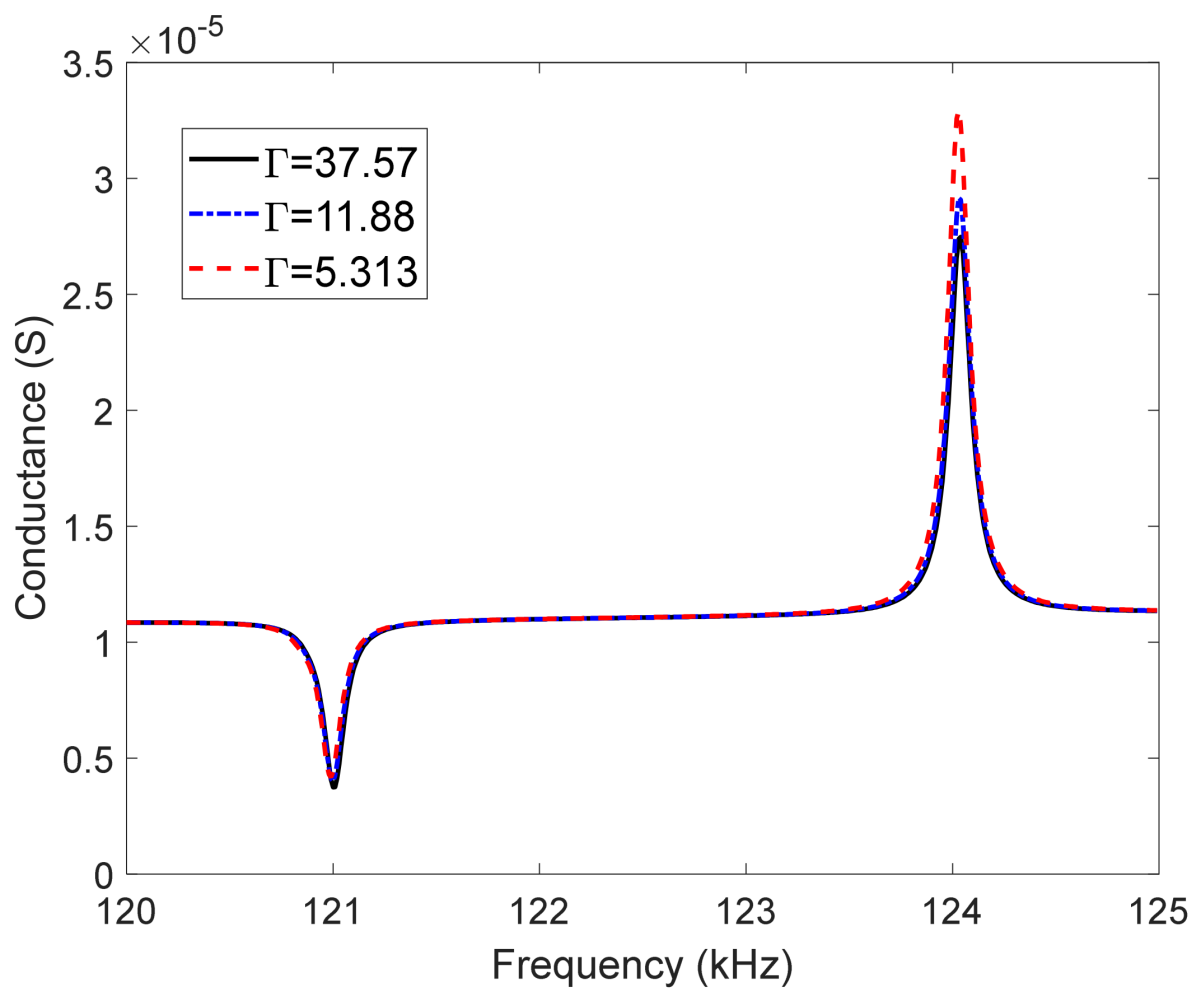
The effect of the bonding adhesive on the cross-admittance signature.

A steel beam measuring 500 mm × 30 mm × 16 mm, as illustrated in Figure 4, was also utilized to evaluate crack localization based on the proposed model. A crack with a width of 1.5 mm and a depth of 8 mm was introduced into the beam to simulate structural damage. The proposed model incorporated the effect of adhesive properties on the interfacial behavior. To investigate the influence of bonding conditions on cross-electromechanical admittance (EMA) signatures, different shear lag parameters were introduced in the analysis. As shown in Figure 8, the shear lag parameter, which directly characterizes the bonding quality, is found to have a minor influence on the cross-EM admittance spectra. Specifically, the peaks of the conductance curve shift slightly toward lower frequencies as the shear lag parameter decreases. This trend aligns well with the behavior reported in Ref. [34] for conventional drive-point EMA signatures. In the case of a sufficiently high shear lag parameter Γ = 37.57, the PZT patch can be regarded as perfectly bonded [28], and this assumption is adopted in the subsequent analysis.

**Figure 9:**
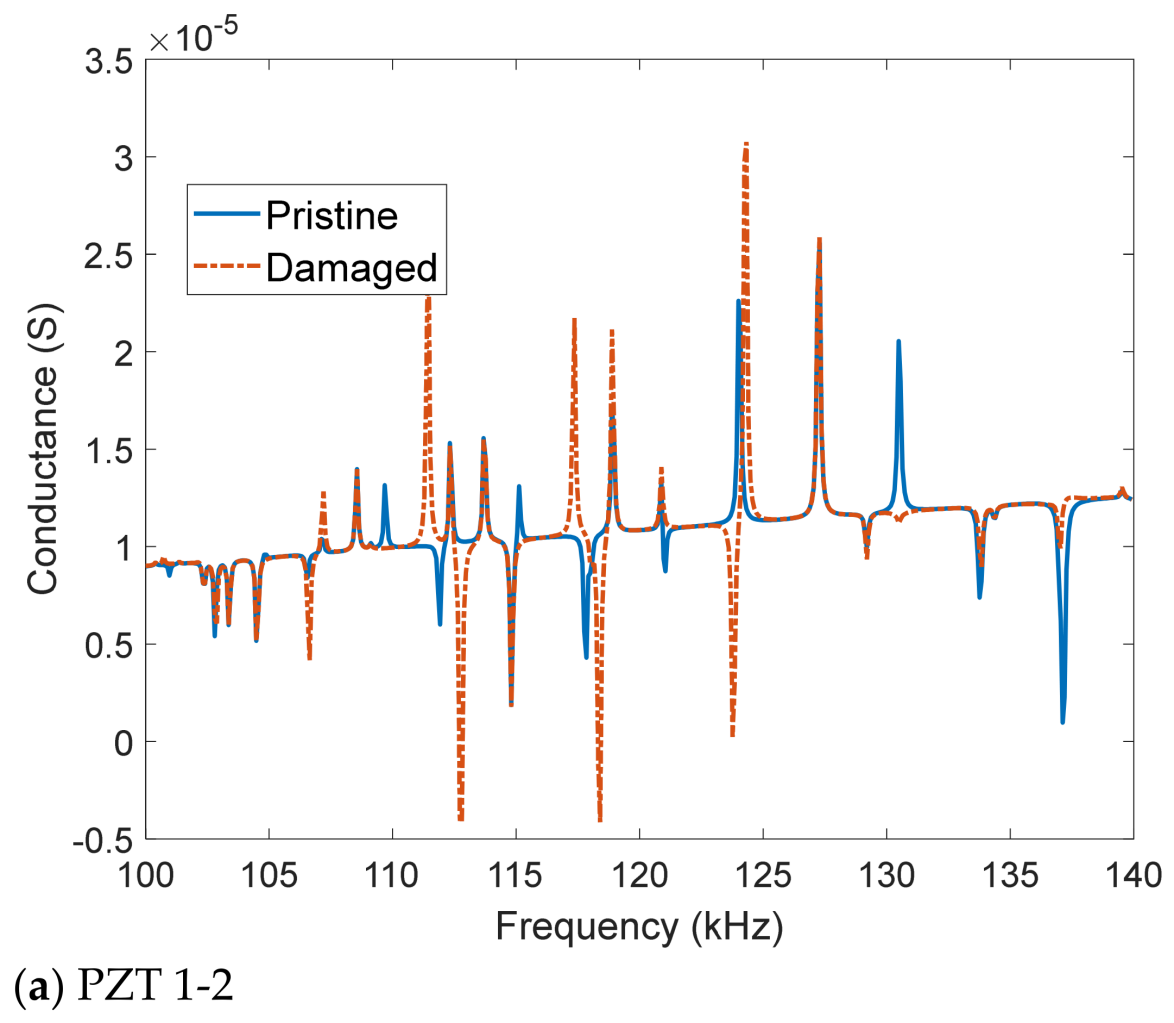

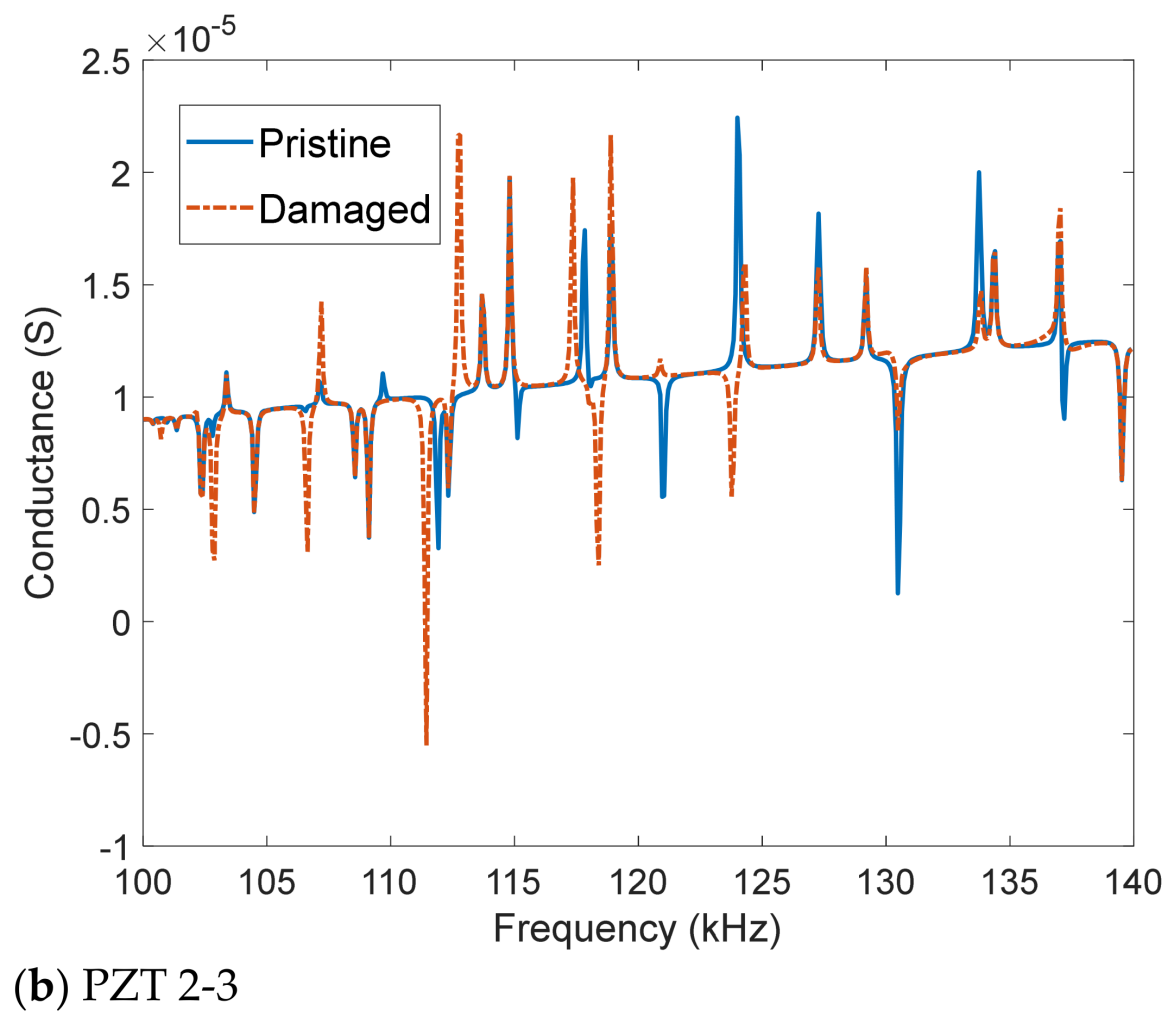
Simulated cross-EM conductance for pristine and cracked beams (case 1).

Two damage configurations were examined based on the present model for crack localization: one with a crack between PZT3 and PZT4, and the other between PZT4 and PZT5. Due to the enhanced sensitivity of high-frequency electromechanical impedance to structural damage, the analysis was conducted in the 100–140 kHz frequency range. The simulated cross-EMA signatures for the first case, obtained under both pristine and damaged conditions, are presented in Figure 9. It should be noted that only two representative datasets were presented for the sake of brevity. It can be observed that the conductance curves reveal distinct damage-induced features, including significant frequency shifts and peak amplitude variations. These conductance spectrum deviations indicate the appearance of structural damage.

**Figure 10:**
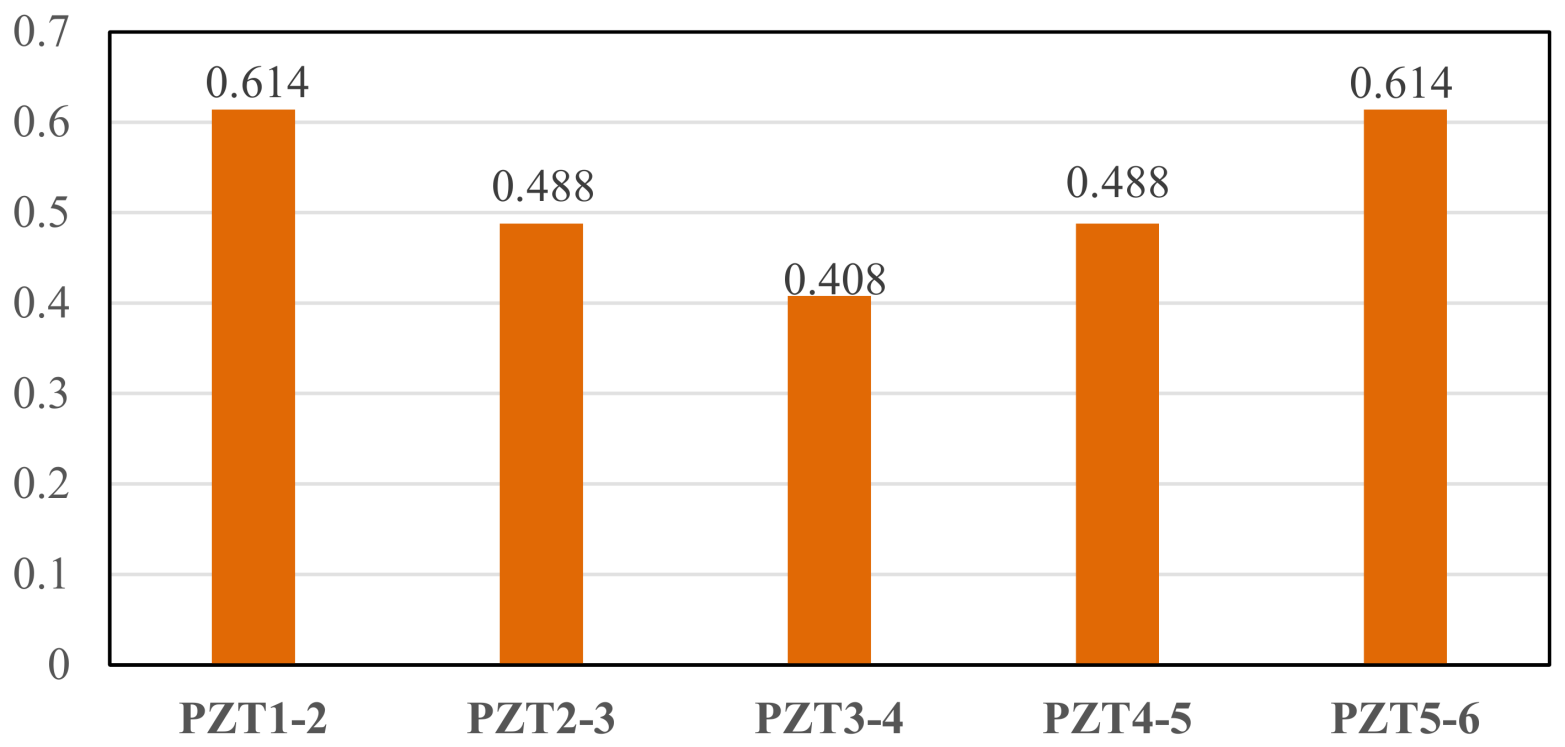
Damage index CC for damage localization via simulated cross EMA (case 1).

**Figure 11:**
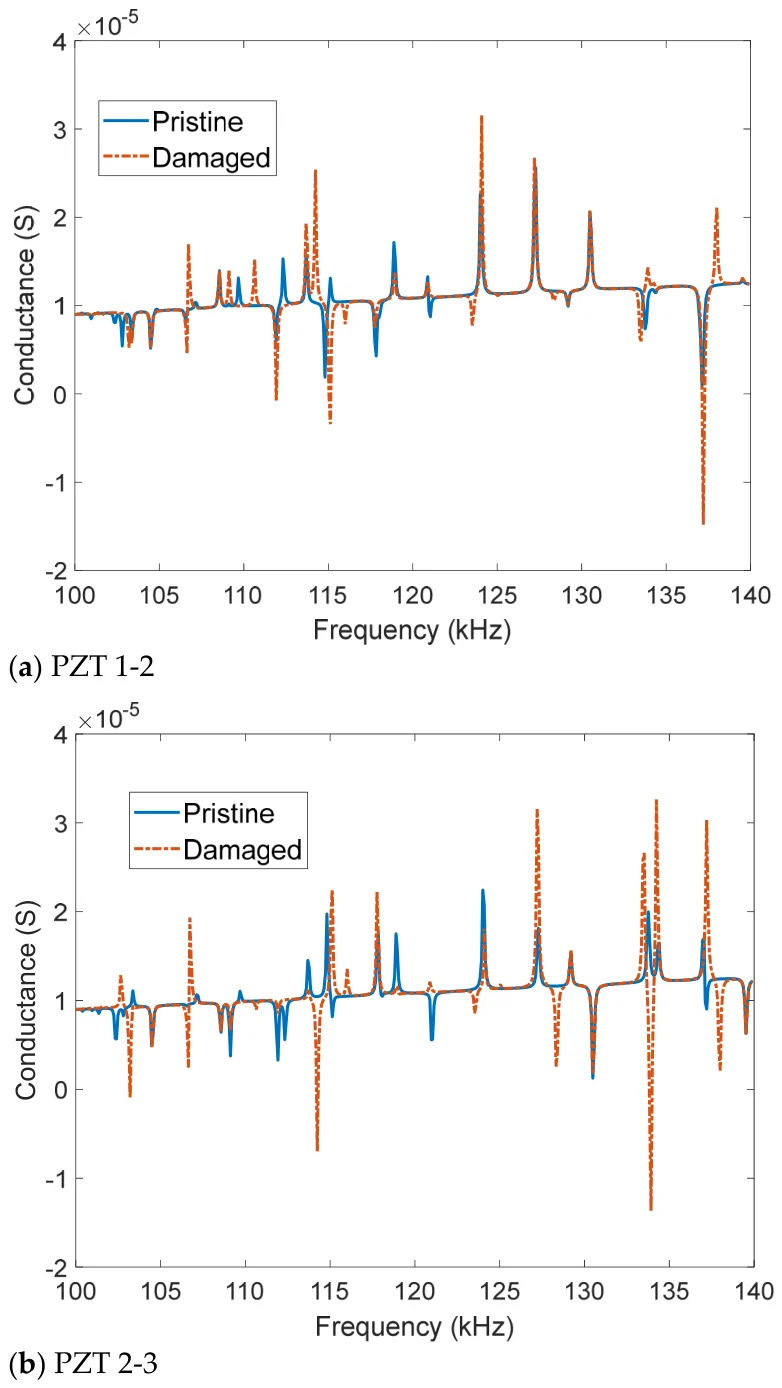
Simulated cross-EM conductance for pristine and cracked beams (case 2).

**Figure 12:**
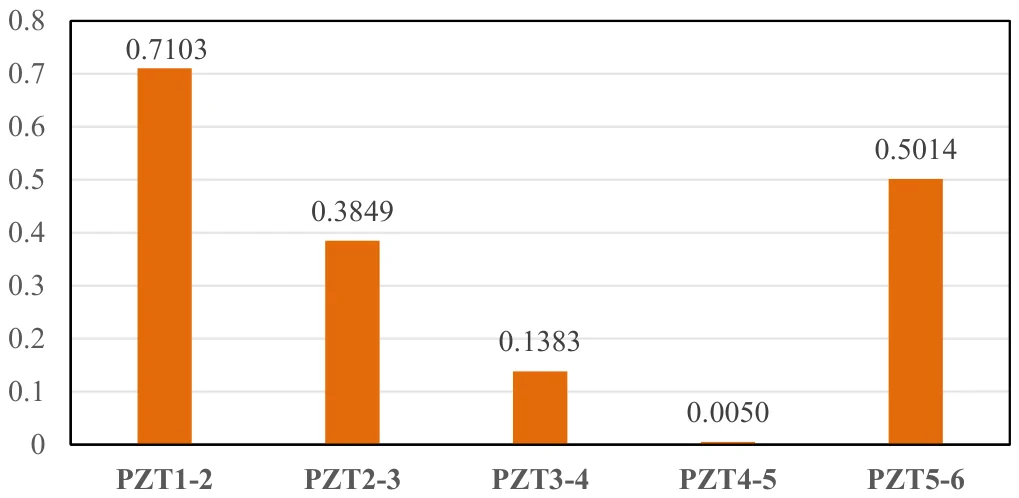
Damage index CC for damage localization via simulated cross EMA (case 2).

However, precise damage localization requires further quantitative analysis through statistical damage indices, including the correlation coefficient (CC) [22,23,24] and Root Mean Square Deviation (RMSD) [35,36,37,38,39,40]. In this study, CC was employed due to its superior advantage in simultaneous evaluation of magnitude–phase relationships and computational efficiency. The calculation formulation for the CC is written as follows [22,23,24]:

(36) $$\mathrm{CC}=\frac{\sum_{i=1}^{N}\left(x_{i}-\bar{x})(y_{i}-\bar{y}\right)}{\sqrt{\sum_{i=1}^{N}{\left(x_{i}-\bar{x}\right)}^{2}\cdot \sum_{i=1}^{N}{\left(y_{i}-\bar{y}\right)}^{2}}},$$

where *x_i_* and *y_i_* are EM conductance data obtained from FEM-based simulation in pristine and damaged states; $\bar{x}$ and $\bar{y}$ are the average EM conductance values of *x_i_* and *y_i_*, respectively. A smaller CC value indicates a weaker correlation between the pristine and damaged conditions. Because the piezoelectric conductance is more sensitive to the local structural defects in their immediate vicinity, the damage location can be approximately determined according to the magnitude of the CC value. Then, the cross-EM data were quantified by the CC damage index for damage localization, as shown in Figure 10. According to the CC damage index, it can be found that the specimen has an abnormally significant value, corresponding to the A3-4 region. This result was completely consistent with the actual location of the damage, indicating that the cross-EM admittance signatures based on the present model can identify and locate the sensor interval containing damage. For the second case, similar findings can be obtained from Figure 11 and Figure 12.

## 4. Crack Localization Using the Experimental Measurements

**Figure 13:**
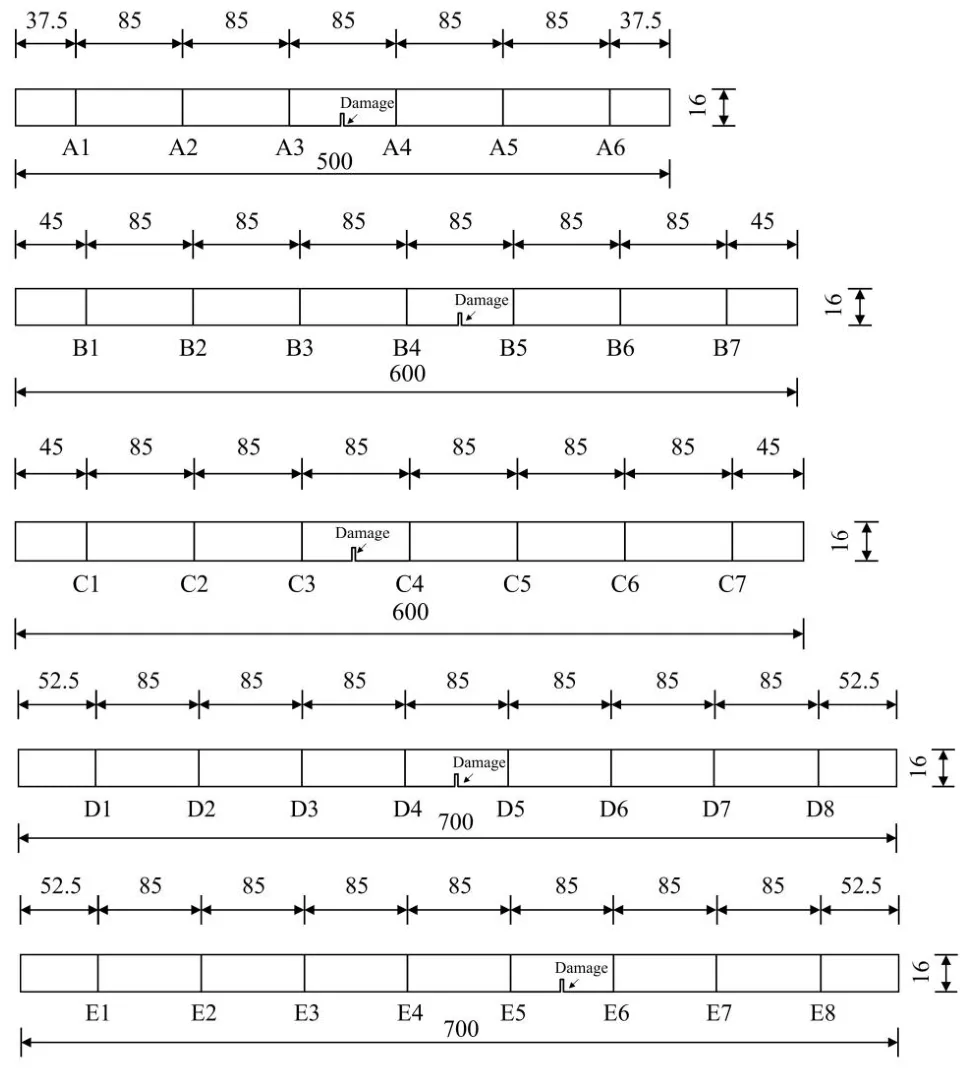
Schematic diagram of steel specimens with crack damage.

In this section, the effectiveness and accuracy of cross admittance for crack localization were further verified experimentally. The investigation employed piezoelectric transducers surface-bonded to five steel specimens, which were divided into three distinct size groups: one 500 mm × 30 mm × 16 mm (Specimen A), two 600 mm × 30 mm × 16 mm (Specimens B and C), and two 700 mm × 30 mm × 16 mm (Specimens D and E), as shown in Figure 13. The specimens were surface-bonded with six (A1-A6), seven (B1-B7 and C1-C7), and eight (D1-D8 and E1-E8) piezoelectric transducers, respectively, arranged with end clearances of 37.5 mm (Specimen A), 45 mm (Specimens B and C), and 52.5 mm (Specimens D and E), maintaining a consistent 85 mm center-to-center spacing (see Figure 13). A rectangular crack of width 1.5 mm and depth 8 mm was introduced by cutting a small slot on each specimen in the beam segment, A3-A4, B4-B5, C3-C4, D4-D5, and E5-E6, to simulate structural damage. In order to reduce the influence of external factors on the experimental results, the steel specimen was placed on the foam plate to approximate the free boundary conditions. During experimental testing, the impedance analyzer interfaces with the piezoelectric transducers via a fixture assembly, forming a closed-loop measurement circuit through simultaneous connection to both positive and negative electrodes of the PZT patch for electromechanical admittance quantification, as shown in Figure 5.

**Figure 14:**
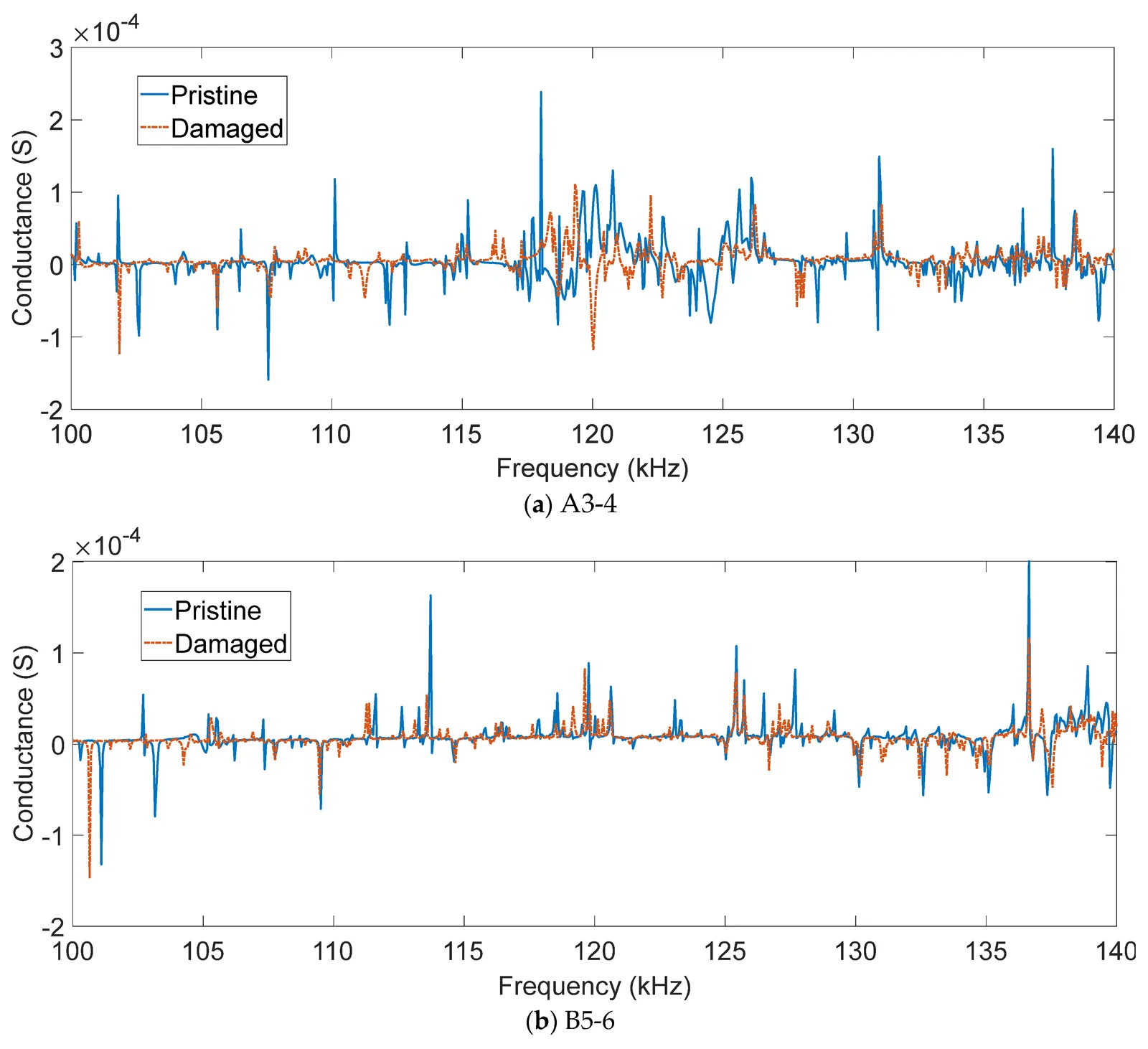

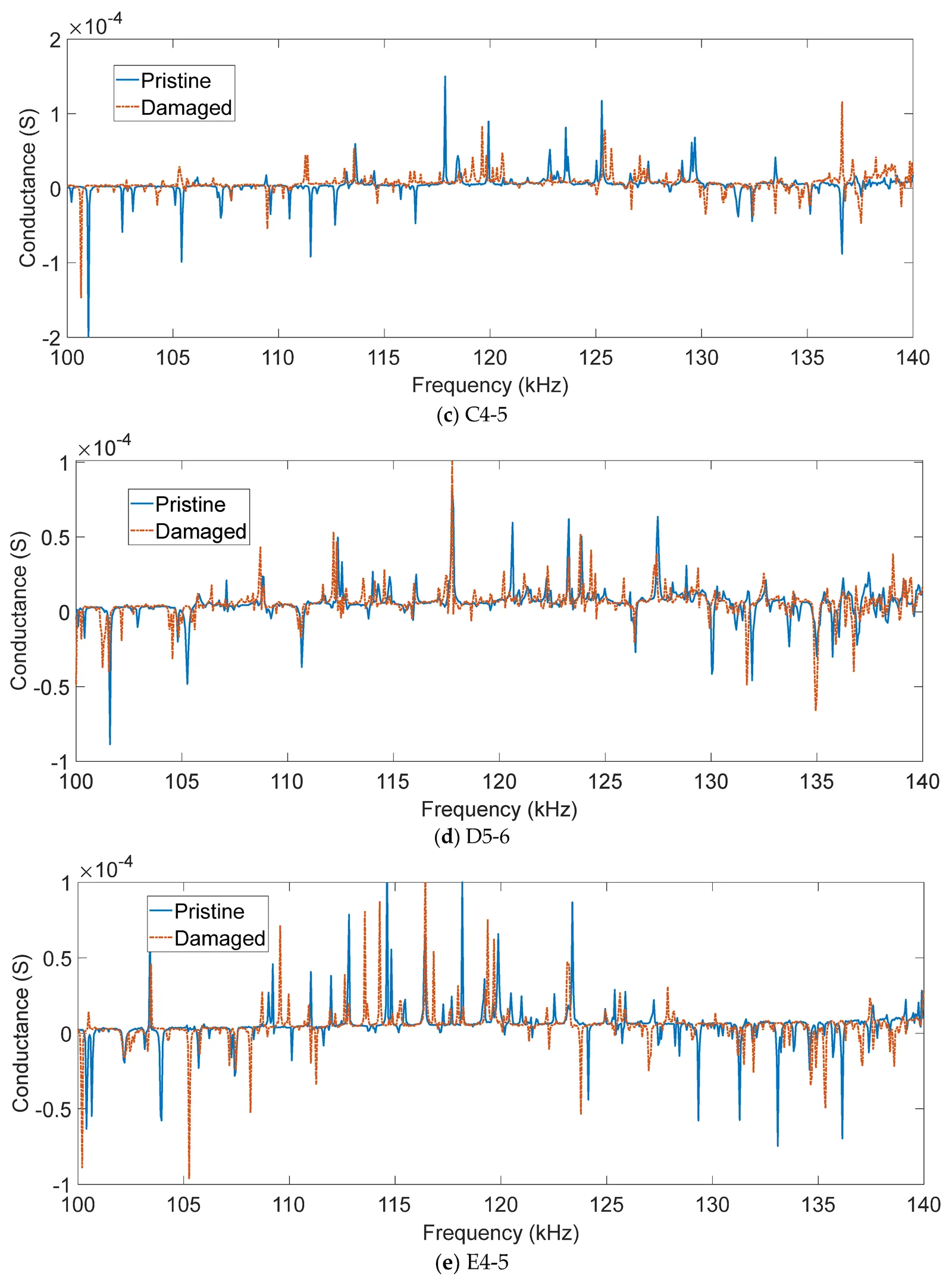
Cross-EMA curves for pristine and damaged beams based on experimental results.

First, the drive-point admittance of each piezoelectric patch was measured. Following this, the group piezoelectric admittance was obtained by applying a high-frequency voltage to two patches connected in parallel, and, finally, the cross admittance was calculated using Equation (32). From Figure 14, it can be seen that the cross-admittance curve in the damaged state changes significantly compared with that in the pristine state. This shows that by comparing the cross-admittance signal spectrum before and after the structural damage, the existence of damage can be qualitatively detected. It should be noted that only one set of cross-EMA curves was depicted for each specimen for the sake of brevity. Compared with the simulated conductance curves shown in Figure 9 and Figure 11, experimental testing yields a larger number of resonant peaks. A similar phenomenon has been reported for cross-EMA measurements on bolted joint structures [41]. Two main sources contribute to this discrepancy. First, high-frequency excitation activates densely distributed modes as well as secondary local modes such as width-wise vibration. Since the present analytical model is built upon Timoshenko’s beam theory, only global flexural and axial motions are considered, and these local width–related vibration modes cannot be reproduced. Second, uniform material properties (mass density, mechanical damping factor, and elastic modulus) are assumed in the analytical formulation, while real specimens exhibit spatially non-uniform material characteristics [41], whose influences are magnified under high-frequency excitation. Theoretically, the governing equations of Timoshenko’s beam theory–based model have no inherent upper frequency limit for global, beam-type responses. However, its accuracy deteriorates when abundant local vibration effects beyond beam assumptions are excited. Within the 100–140 kHz band adopted for crack localization, the proposed model can capture the trend of cross-EMA spectral variations caused by transverse beam cracks, but full quantitative agreement with experimental resonant peaks is not guaranteed.

**Figure 15:**
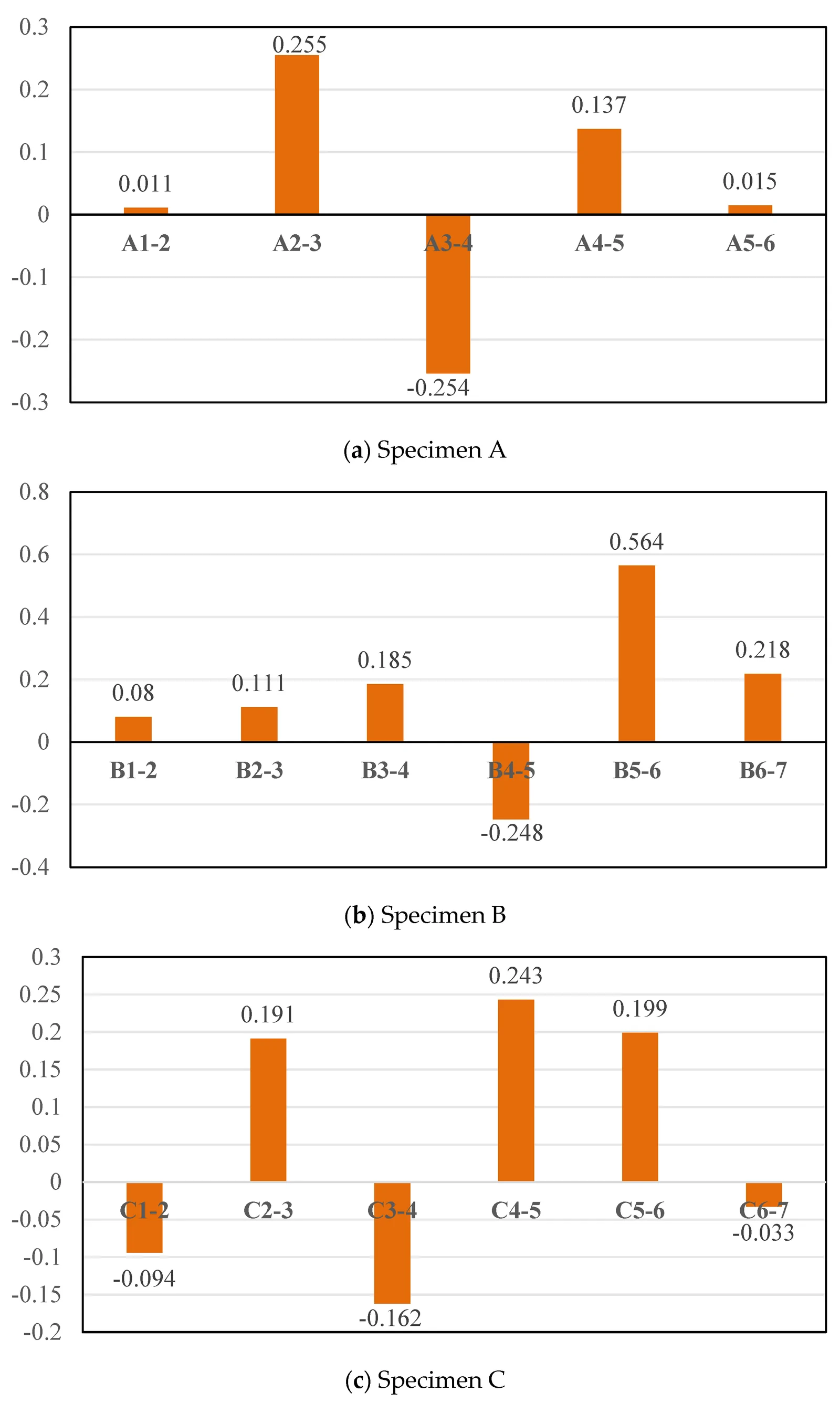

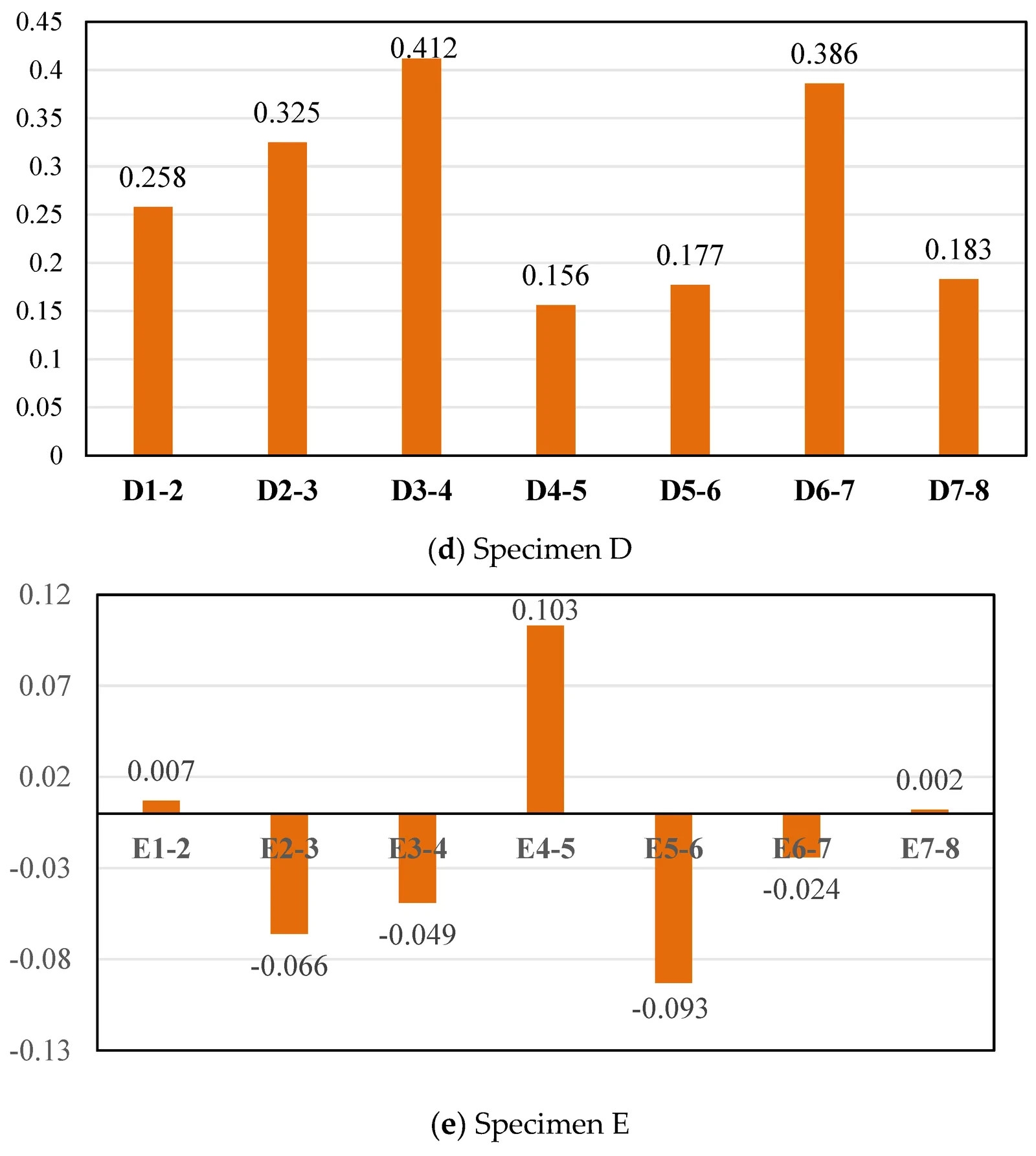
Damage index CC for damage localization based on experimental cross-EM conductance.

In order to determine the location of the damage, we introduce the statistical damage index CC to process the cross-admittance signal data for damage location analysis. Figure 15 demonstrates that the minimum value of the CC index of each test specimen corresponds to the A3-4, B4-5, C3-4, D4-5, and E5-6 areas, respectively. In this single measurement, the minimum CC value falls within the damaged segment for each specimen. This confirms that the CC statistical index based on cross EMA is capable of identifying the sensor interval containing damage for beam components under laboratory test conditions. It is worth noting the considerable discrepancy between simulated and experimental CC indices. Simulated CC values for damaged beam segments remain consistently positive (see Figure 10 and Figure 12), while many experimental CC values corresponding to damaged intervals become negative. This observation indicates that the proposed analytical model underestimates the extent of spectral distortion in cross-EMA signatures induced by crack damage under 100–140 kHz excitation. Multiple physical factors collectively lead to this mismatch. First, the crack is simplified as a rotational spring within the analytical model, which only accounts for local rotational flexibility and fails to fully capture complex local stress redistribution, contact-induced micro-vibration, and crack-surface interactions present in real, machined slots. Second, frequency-independent damping is adopted for steel, PZT patches, and adhesive layers in the simulation. In reality, material and interfacial damping are frequency-dependent, and higher damping loss at high frequencies will produce stronger spectral modification that is not fully reproduced by the model. Third, although adhesive parameters are incorporated in the TMM formulation, the model assumes uniform and ideal bonding conditions. In practical specimens, minor spatial inhomogeneities of bond layer thickness and local interfacial imperfections can further distort measured cross-EMA spectra. Fourth, as discussed previously, high-frequency excitation excites additional local width-wise vibration modes, which are excluded from Timoshenko’s beam theory–based analytical framework. Notwithstanding this quantitative mismatch in the magnitude and sign of CC values, both the simulations and experiments consistently place the minimum CC index within the actual damaged segment. Accordingly, even though the proposed model cannot precisely predict the exact magnitude or sign of experimental CC values, it reliably captures the relative trend of reduced CC values over the sensor interval containing damage.

## 5. Conclusions

This study developed a theoretical model to analyze the cross-electromechanical admittance (EMA) response of cracked beams. Numerical simulations showed close agreement with both experimental and finite element results, confirming the model’s reliability for damage localization. Moreover, the combination of simulated cross-admittance signatures with the correlation coefficient (CC) index demonstrated promising potential for structural damage identification. Experimental validation further confirmed the feasibility of the cross-admittance approach for localizing the introduced cracks. This technique, which involves connecting two piezoelectric patches in parallel, effectively captures the local dynamic characteristics between them. Consequently, under laboratory conditions, it can identify the sensor interval containing cracks and offers promising prospects for structural health monitoring applications.

The proposed cross-EMA approach represents a meaningful extension of the conventional drive-point admittance technique. The synergistic integration of both EMA methods provides complementary information, resulting in a more comprehensive and accurate damage identification methodology. Furthermore, the use of cross EMA extends the technical tools available for structural health monitoring based on the EMA technique, offering enhanced testing capabilities for damage detection.

## Figures and Tables

**Table 1:** Material constants and geometric parameters of PZT patch [29].

*E_p_* (N/m^2^)	*η*	*ρ_p_* (kg/m^3^)	*d*_31_ (m/V)	$\varepsilon_{33}^{T}$(F/m)	*δ*	Geometry (mm^3^)
6.67 × 10^10^	0.03	7800	−2.10 × 10^−10^	2.14 × 10^−8^	0.0185	15 × 15 × 0.2

## Data Availability

The data presented in this study are available from the corresponding author upon reasonable request.

## Nomenclature

*a*	Half-length of PZT patch
*c*	Depth of crack
*d*_31_	Piezoelectric constant
*f*	Frequency
*h_p_*, *h_a_*, *h_s_*	Thickness of piezoelectric patch, bonding layer, and host beam, respectively
*i*	$\sqrt{-1}$
*l*	Length of host beam
*l_p_*,	Length of PZT wafer
*t*	Time variable
*u*	Axial displacement due to axial force in host beam
*u_p_*	Axial displacement of PZT patch
*u_s_*	Axial displacement on the surface of the host beam
*w*	Transverse deflection
*w_p_*	Width of PZT patch
*x*, *y*, *z*	Cartesian coordinates
*A*	Cross-sectional area
*D*_3_	Electric displacement
*E*_3_	Electric potential
*E_p_*,* E_s_*	Young’s modulus of PZT patch and host beam, respectively
*η*	Mechanical loss factors
${\bar{E}}_{p}$	Complex Young’s modulus of PZT patch, ${\bar{E}}_{p}=E_{p}(1+\eta i)$
*G_a_*, *G*	Shear rigidity of bonding layer and host beam, respectively
*I*	Moment of area
*I_i_*	Electric current flowing through *i*-th PZT sensor
*M*, *N*, *Q*	Bending moment, axial force, and shear force, respectively
*T*_1_	Longitudinal stress in PZT patch
*V*	Electric voltage
*Y*	Electric admittance of PZT patch
*δ*	Dielectric loss factors
$\varepsilon_{33}^{T}$, ${\bar{\varepsilon }}_{33}^{T}$	Dielectric constant and complex dielectric constant, respectively, and ${\bar{\varepsilon }}_{33}^{T}=\varepsilon_{33}^{T}(1-\delta i)$
*ε_p_*	Axial strain of PZT patch
*φ*	Shear rotation
*κ*	Shear correction factor (*π*^2^/12)
*μ*	Poisson’s ratio of host structural member
*ρ_p_*, *ρ_s_*	Mass density of PZT patch and host beam, respectively
*τ(x)*	Transferred shear stress between PZT patch and the host beam
*ω*	Circular frequency
*ψ*	Stiffness ratio, *ψ* = *E_s_h_s_*/(*E_p_h_p_*)
Γ	Shear lag parameter $\Gamma =\sqrt{(\bar{G}\theta_{a}/{\bar{h}}_{a}^{2})(\psi +\alpha )/\psi }$, where $\bar{G}=G_{a}/E_{p}$, *θ_a_* = *h_a_*/*h_p_*, ${\bar{h}}_{a}=h_{a}/a$, *α* = 1.
